# Comparative impact of pharmacological treatments for gestational diabetes on neonatal anthropometry independent of maternal glycaemic control: A systematic review and meta-analysis

**DOI:** 10.1371/journal.pmed.1003126

**Published:** 2020-05-22

**Authors:** Jane L. Tarry-Adkins, Catherine E. Aiken, Susan E. Ozanne

**Affiliations:** 1 Metabolic Research Laboratories and MRC Metabolic Diseases Unit, Wellcome Trust-MRC Institute of Metabolic Science, University of Cambridge, Cambridge, United Kingdom; 2 Department of Obstetrics and Gynaecology, the Rosie Hospital and NIHR Cambridge Comprehensive Biomedical Research Centre, University of Cambridge, Cambridge, United Kingdom; University of Bristol School of Clinical Science, UNITED KINGDOM

## Abstract

**Background:**

Fetal growth in gestational diabetes mellitus (GDM) is directly linked to maternal glycaemic control; however, this relationship may be altered by oral anti-hyperglycaemic agents. Unlike insulin, such drugs cross the placenta and may thus have independent effects on fetal or placental tissues. We investigated the association between GDM treatment and fetal, neonatal, and childhood growth.

**Methods and findings:**

PubMed, Ovid Embase, Medline, Web of Science, ClinicalTrials.gov, and Cochrane databases were systematically searched (inception to 12 February 2020). Outcomes of GDM-affected pregnancies randomised to treatment with metformin, glyburide, or insulin were included. Studies including preexisting diabetes or nondiabetic women were excluded. Two reviewers independently assessed eligibility and risk of bias, with conflicts resolved by a third reviewer. Maternal outcome measures were glycaemic control, weight gain, and treatment failure. Offspring anthropometric parameters included fetal, neonatal, and childhood weight and body composition data. Thirty-three studies (*n* = 4,944), from geographical locations including Europe, North Africa, the Middle East, Asia, Australia/New Zealand, and the United States/Latin America, met eligibility criteria. Twenty-two studies (*n* = 2,801) randomised women to metformin versus insulin, 8 studies (*n* = 1,722) to glyburide versus insulin, and 3 studies (*n* = 421) to metformin versus glyburide. Eleven studies (*n* = 2,204) reported maternal outcomes. No differences in fasting blood glucose (FBS), random blood glucose (RBS), or glycated haemoglobin (HbA1c) were reported. No studies reported fetal growth parameters. Thirty-three studies (*n* = 4,733) reported birth weight. Glyburide-exposed neonates were heavier at birth (58.20 g, 95% confidence interval [CI] 10.10–106.31, *p* = 0.02) with increased risk of macrosomia (odds ratio [OR] 1.38, 95% CI 1.01–1.89, *p* = 0.04) versus neonates of insulin-treated mothers. Metformin-exposed neonates were born lighter (−73.92 g, 95% CI −114.79 to −33.06 g, *p* < 0.001) with reduced risk of macrosomia (OR 0.60, 95% CI 0.45–0.79, *p* < 0.001) than insulin-exposed neonates. Metformin-exposed neonates were born lighter (−191.73 g, 95% CI −288.01 to −94.74, *p* < 0.001) with a nonsignificant reduction in macrosomia risk (OR 0.32, 95% CI 0.08–1.19, I_2_ = 0%, *p* = 0.09) versus glyburide-exposed neonates. Glyburide-exposed neonates had a nonsignificant increase in total fat mass (103.2 g, 95% CI −3.91 to 210.31, *p* = 0.06) and increased abdominal (0.90 cm, 95% CI 0.03–1.77, *p* = 0.04) and chest circumferences (0.80 cm, 95% CI 0.07–1.53, *p* = 0.03) versus insulin-exposed neonates. Metformin-exposed neonates had decreased ponderal index (−0.13 kg/m^3^, 95% CI −0.26 to −0.00, *p* = 0.04) and reduced head (−0.21, 95% CI −0.39 to −0.03, *p* = 0.03) and chest circumferences (−0.34 cm, 95% CI −0.62 to −0.05, *p* = 0.02) versus the insulin-treated group. Metformin-exposed neonates had decreased ponderal index (−0.09 kg/m^3^, 95% CI −0.17 to −0.01, *p* = 0.03) versus glyburide-exposed neonates. Study limitations include heterogeneity in dosing, heterogeneity in GDM diagnostic criteria, and few studies reporting longitudinal growth outcomes.

**Conclusions:**

Maternal randomisation to glyburide resulted in heavier neonates with a propensity to increased adiposity versus insulin- or metformin-exposed groups. Metformin-exposed neonates were lighter with reduced lean mass versus insulin- or glyburide-exposed groups, independent of maternal glycaemic control. Oral anti-hyperglycaemics cross the placenta, so effects on fetal anthropometry could result from direct actions on the fetus and/or placenta. We highlight a need for further studies examining the effects of intrauterine exposure to antidiabetic agents on longitudinal growth, and the importance of monitoring fetal growth and maternal glycaemic control when treating GDM. This review protocol was registered with PROSPERO (CRD42019134664/CRD42018117503).

## Abstract 2

Jane Tarry-Adkins and colleagues reveal the anthropometric impact of pharmacological treatments for gestational diabetes on babies.

## Author summary

**Why was this study done?:**

- Gestational diabetes mellitus (GDM) is an increasing healthcare concern that has significant short- and long-term health implications for mother and baby.
- Optimising clinical treatment of GDM is an important priority. Several current treatment options exist, including insulin or oral therapies, such as metformin or glyburide.
- We aimed to investigate the associations between different GDM treatments and the growth of the baby in the womb, at birth, during childhood, and in later life.

**What did the researchers do and find?:**

- We performed a systematic review of 33 studies that included 4,944 mothers who were randomised to insulin, metformin, or glyburide for treatment of GDM. We included all studies that reported the weight and growth of their babies in the womb, at birth, or later in childhood.
- Babies exposed to glyburide are significantly heavier at birth compared to those whose mothers were randomised to insulin or metformin. Conversely, metformin-exposed babies are significantly lighter at birth than those whose mothers were randomised to insulin or glyburide.
- There may also be differences in body composition at birth. Babies exposed to glyburide tend to be born larger with increased fat mass, and babies exposed to metformin were born smaller and were thinner, compared to those treated with insulin.

**What do these findings mean?:**

- There are significant differences in body mass between babies whose mothers were randomised to glyburide, metformin, and insulin to treat GDM. Our results highlight the importance of considering the effects of treatment on both mother and baby when managing GDM.
- There is a need for better understanding of exactly how oral treatments for GDM impact on growth of babies in the womb and in later life, particularly whether there are implications for long-term health.

## Introduction

Gestational diabetes mellitus (GDM) is an increasing healthcare concern, affecting 3% to 25% of pregnancies worldwide [1]. It was estimated in 2017 that approximately one in seven babies globally was born to a mother with GDM [2]. GDM has significant short- and long-term health implications for mother and baby, particularly if untreated or undertreated, making optimal clinical management of GDM an important priority. Poorly managed or untreated GDM leads to accelerated fetal growth [3] and therefore increases the risk of macrosomic and large-for-gestational-age neonates [4,5]. Pregnancies affected by GDM that are not adequately managed are consequently at risk of adverse neonatal outcomes, both immediately; e.g., shoulder dystocia, birth trauma, including birth hypoxic injuries, and neonatal hypoglycaemia [6–8], and in the longer term; e.g., metabolic dysregulation in later childhood [9,10]. Hence, it is essential to implement effective clinical interventions to maintain maternal glycaemic control, with the dual aim of maintaining fetal growth within normal parameters, thereby protecting both mother and baby from adverse outcomes.

In around two thirds of GDM-affected pregnancies, maternal euglycaemia can be maintained by implementing strategies for altering diet and lifestyle [11]. However, at least one third of affected women will require pharmacological therapies to achieve their treatment goals [12]. Three commonly used pharmacological options are available: insulin, metformin, and glyburide (also known as glibenclamide). However, there is little international consensus on optimal management strategies, in particular, which pharmacological agent(s) should be offered as first-line therapies. The endogenous hormone insulin [13] is recommended as the first-line treatment for GDM by several organisations, including the American Diabetes Association (ADA) [14], the American College of Obstetrians and Gynecologists (ACOG) [15] and the Canadian Diabetes Association (CDA) [16]. Metformin (N,N-dimethylbiguanide), a biguanide oral insulin sensitiser and glucose-lowering drug, is recommended by the Society of Maternal-Fetal Medicine (SMFM) as a first-line treatment for GDM [17]. The United Kingdom’s regulatory body, the National Institute for Clinical Excellence (NICE) recommends metformin for use in GDM as an adjunct or alternative to insulin in GDM management [18]. New Zealand’s regulatory body also recommends metformin for the treatment of GDM [19]. Glyburide (5-chloro-N(2-{4-[N-(N-cyclohexylcarbomoyl)sulfamoyl]phenyl}ethyl)-2-methoxybenzamide) belongs to the sulphonylurea class of antidiabetic agents, which stimulates insulin secretion from pancreatic beta cells, thereby reducing hyperglycaemia. Both the ADA [14] and the ACOG [15] consider its use acceptable in GDM. In the UK, NICE guidelines suggest considering glyburide only for women with gestational diabetes who do not respond to or cannot tolerate metformin but decline insulin [18]. However, the Food and Drug Administration (FDA) classifies glyburide as a Category C class drug (not approved in GDM), and in Australia, the Royal Australian College of General Practitioners (RACGP) [20] suggests it should be used with caution. Given the range of international opinion, there is a need to fully understand the risks and benefits of each option, not only for the mother but also for the developing fetus, in the short and long term.

The dual goals of clinical GDM management (achieving maternal euglycaemia and maintaining normal fetal growth trajectory) are often assumed to be directly linked. This is the case if GDM is controlled with dietary modification and also if using insulin, which does not cross the placenta [21], although it is possible that insulin could have additional effects at the materno-fetal interface. However, the commonly used oral anti-hyperglycaemic agents metformin and glyburide both cross the placenta to varying degrees. Recent work shows umbilical cord serum concentrations of metformin at the time of delivery are comparable to or exceed maternal concentrations [22–24], and it is present at clinically relevant concentrations in fetal and placental tissues (50%–100% of maternal concentrations) [22,23]. Studies have also shown placental transfer of glyburide, with concentrations of glyburide in umbilical cord plasma approximately 70% of maternal levels [25–27]. These findings suggest that there is potential for both metformin and glyburide to exert effects on the developing fetus and on the placenta via direct or indirect pathways, independent of maternal glycaemic control.

Hence, while fetal growth can be assumed to be directly indexed to maternal glucose levels in GDM treated with diet or insulin, there is the possibility of the uncoupling of maternal glycaemic control and fetal growth as a result of treatment with metformin or glyburide. The aim of this study was therefore to provide a systematic, unbiased, and comprehensive overview of the comparative impacts of various pharmacological treatments for GDM on fetal growth, neonatal anthropometry, and childhood growth outcomes, including considering the effects of maternal glycaemic control.

## Materials and methods

This systematic review and meta-analysis was conducted in accordance with the Preferred Reporting Items for Systematic Reviews and Meta-Analyses (PRISMA) guidelines [28]. The PRISMA checklist is detailed in S1 PRISMA Checklist. The systematic review protocol was registered in PROSPERO CRD42019134664 (S1 Text). Metformin versus insulin data were derived from PROSPERO protocol CRD42018117503 [29]. Ethical approval was not required for this meta-analysis.

### Literature searches, search strategies, and eligibility criteria

Systematic literature searches using prespecified terms (S2 Text) were performed on PubMed (June 1997 to 12 February 2020), Ovid EMBASE (1974 to 12 February 2020), Ovid Medline (1946 to 12 February 2020), Cochrane Library (database inception to 12 February 2020), Clinicaltrials.gov (database inception to 12 February 2020), and Web of Science (1900 to 12 February 2020). Details for the database search strategies for metformin versus insulin comparisons can be found in [29]. No language or location restrictions were applied. Studies that randomised women with GDM to glyburide versus insulin therapy, metformin versus insulin therapy, and metformin versus glyburide therapy were included. Studies were excluded if they investigated other oral anti-glycaemic agents (such as myo-inositol), or if interventions were given prior to pregnancy. GDM was screened for and diagnosed according to local criteria in each study, and we did not apply exclusions with respect to this. Studies were excluded if they included participants with multiple pregnancies or preexisting diabetes, or if they randomised fewer than 50 women in total. Studies were also excluded if they were not analysed on an intention-to-treat (ITT) basis. Studies were excluded if trial participants were omitted from the study on the basis of fetal weight and/or birth weight. Data reported only in meeting abstracts would have been included if the abstract contained sufficient information for assessment, but none met this criterion. Where insufficient information for assessment was available, authors were contacted for further information. Three studies provided insufficient information for assessment; however, none of these authors responded to contact, and therefore these studies were not included in the analysis.

### Study selection and data extraction

**Fig 1:**
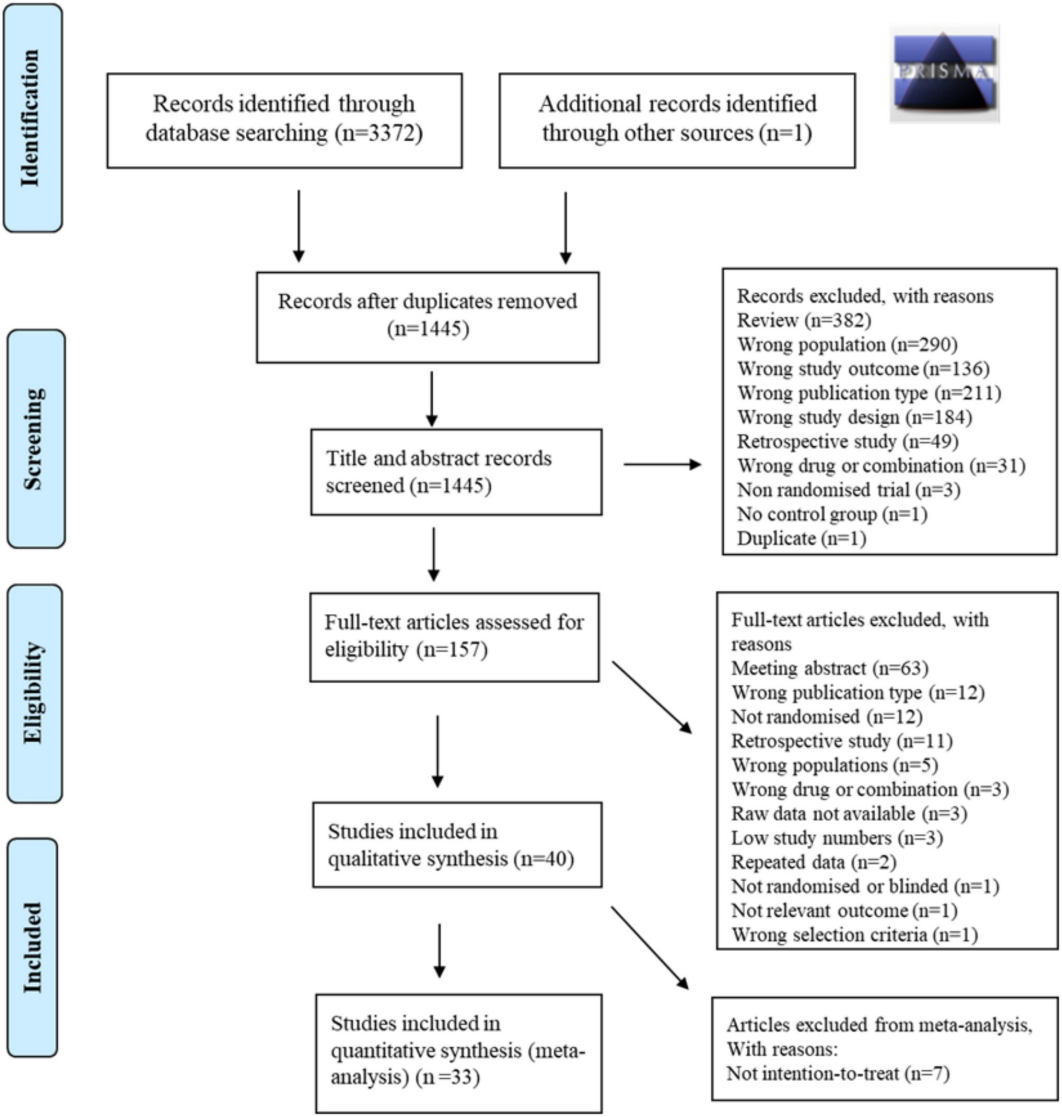
PRISMA flow diagram (metformin versus insulin, glyburide versus insulin, glyburide versus metformin). PRISMA, Preferred Reporting Items for Systematic Reviews and Meta-Analyses.

Two reviewers (JLA and CEA) independently assessed each study using predetermined inclusion/exclusion criteria (detailed in S1 Table). A third reviewer (SEO) was available to resolve cases for which eligibility was unclear. An initial screen of titles and abstracts was performed, followed by a detailed full paper screen (Fig 1).

Data extraction from eligible studies was conducted independently using a standardised proforma by two authors (JLA and CEA). Fetal and neonatal outcome measures were as follows: fetal growth parameters (head circumference, abdominal circumference, femur length, biparietal diameter, estimated fetal weight calculated by any formula), birth weight (g or kg), large for gestational age (LGA: birth weight >90th percentile for gestational age), macrosomia (birth weight >4 kg), neonatal ponderal index (kg/m^3^), neonatal abdominal, head, chest, and waist circumferences (cm), neonatal skinfold thicknesses (mm), and neonatal fat masses (total and abdominal; g). We also collected data on maternal outcomes that reflected the adequacy of GDM control: fasting blood glucose (FBS; mg/dL), random blood glucose (RBS, average 2-hour postprandial glucose measurements; mg/dL), glycated haemoglobin (HbA1c; %), treatment failure rates (defined as per the criteria from each original study), and gestational weight gain (kg). FBS and RBS were measured in the last two weeks of pregnancy by two studies [30,31], and one study did not specify when these measurements were taken [32]. All studies reported that HbA1c values were taken just before delivery. Gestational weight gain was measured throughout pregnancy in most studies [33–42]. Other studies measured this outcome from treatment start until the end of pregnancy [31,34,41–45].

### Quality assessment of included studies (risk of bias in individual studies)

Each study was independently assessed by two authors (JLA and CEA) for quality and validity using the Cochrane Collaboration tool for assessing risk of bias. Seven risk of bias domains were assessed for each study and each domain was given a rating of low risk, unknown risk, or high risk of bias (S2 Table). All risk of bias analysis was conducted at the study level.

The principle summary measures utilised in this systematic review were unadjusted odds ratios (ORs) (for dichotomous data) or differences in means (for continuous data). Meta-analysis was performed using Review Manager (RevMan) Version 5.3, Copenhagen: The Nordic Cochrane Centre, the Cochrane Collaboration, 2014) and the ‘*metafor*’ package in R version 3.5.1 [46]. Funnel plots were constructed to assess publication bias. Meta-analyses with 5 or more studies included were also subjected to Egger’s test. Heterogeneity between studies was assessed using the I-squared statistic, and any outcomes showing significant inter-study heterogeneity were analysed using a random-effects model. Sensitivity analyses were performed using ‘leave-one-out’ (LOO) sensitivity testing for individual studies, ‘leave-one-criteria-out’ sensitivity testing for studies grouped according to GDM criteria, and ‘leave-one-continent-out’ sensitivity analysis for studies grouped according to geographical location [29]. Where *p*-values are reported, an alpha level <0.05 was considered statistically significant.

## Results

For the comparisons of drug treatments for gestational diabetes, electronic searching of the specified databases yielded a total of 3,373 studies. After removal of duplicates and title/abstract screening, 157 trials were screened for full-text assessment, applying the full set of eligibility criteria. After full-text evaluation, a total of 40 studies remained eligible for inclusion. Seven studies were removed due to not being analysed on an ITT basis, leaving 33 studies for meta-analysis (Fig 1). This represented a total 4,944 pregnancies (Table 1). For all comparisons, the studies varied with respect to quality and design (S3 Table). Measured outcomes varied between studies and comparisons, with birth weight the single most commonly reported outcome (Table 1).

**Table 1:** Study and participant numbers.

Study type	Glyburide versus insulin	Metformin versus insulin	Metformin versus glyburide
All included studies	8 studies (*n* = 1,722)	22 studies (*n* = 2,801)	3 studies (*n* = 421)
Fetal growth	0 studies (*n* = 0)	0 studies (*n* = 0)	0 studies (*n* = 0)
Birth weight	7 studies (*n* = 1,651)	11 studies (*n* = 1,820)	3 studies (*n* = 421)
Neonatal anthropometry	8 studies (*n* = 1,722)	12 studies (*n* = 2,590)	3 studies (*n* = 421)
Infant/child growth	0 studies (*n* = 0)	Up to 3 studies (*n* = 986)	0 studies (*n* = 0)

The doses of glyburide (1.25 mg to 20 mg daily) and metformin (500 mg to 3,000 mg daily) demonstrated considerable heterogeneity, both within and between studies. Heterogeneity also existed between studies in criteria used to diagnose GDM, with a total of 9 different diagnostic criteria used. These were the ADA, Australasian Diabetes in Pregnancy Society (ADIPS), Brazilian Health Ministry (BHM), Carpenter-Coustan (CC), Finnish National Criteria (FNC), International Association of Diabetes and Pregnancy (IADPSG), National Diabetes Data Group (NDDG), World Health Organization (WHO), and unspecified GDM criteria. LOO sensitivity analysis demonstrated that use of different thresholds for GDM diagnosis did not have a significant impact on the meta-analyses for birth weight (S1 Fig). In general, there was also a range of geographical settings, including Europe [33,34,47,48–50] (six studies; *n* = 1,528), Australia/New Zealand [43,51,52] (three studies; *n* = 1,259), Latin America [36–39,44] (five studies; n = 483), North Africa/Middle East [30–32,40,41,45] (six studies; *n* = 637), the US [35,53–55] (four studies; *n* = 698), and South East Asia [42,56–59] (five studies; *n* = 1,113). More studies from North Africa/Middle East [30,31,40,41,45] (five studies; *n* = 542) and Europe [33,34,47–49] (five studies; *n* = 719) compared metformin with insulin. More studies from the US/Latin America [35–39,53,54,55] (eight studies; *n* = 1,900) compared glyburide with either insulin or metformin. Leave-one-continent-out sensitivity analysis showed that the difference in birth weight between glyburide versus insulin groups became nonsignificant after leaving out studies from the US/Latin America. Similarly, the difference in birth weight between metformin versus insulin groups became nonsignificant after leaving out studies from North Africa/Middle East [30,31,40,41,45] (S2 Fig). Metformin versus glyburide studies only originated from the US/Latin America, and therefore leave-one-continent out analysis was not conducted.

Overall, the risk of bias was moderate to low in the majority of included studies (S2 Table). We assessed the likelihood of single studies significantly influencing the overall results using leave-one-study out sensitivity analysis. Regarding maternal outcomes, of 13 meta-analyses performed, three were not robust to leave-one-study-out testing (gestational weight gain comparing metformin with insulin; gestational weight gain comparing metformin with glyburide; FBS comparing metformin with insulin) (S3 Fig). Of the nine meta-analyses examining neonatal growth outcomes, three were not robust to leave-one-study-out testing (birth weight comparing glyburide with insulin; LGA comparing metformin with glyburide; macrosomia comparing metformin with insulin). Funnel plots for all outcomes were assessed visually for asymmetry (S4 Fig). Egger’s testing demonstrated no evidence of publication bias in any outcomes or comparisons, with the exception of birth weight for studies comparing glyburide with insulin and for FBS when comparing metformin with insulin (S4 Table).

### Maternal demographics and outcomes

Table 2 reports the demographic data of the participants of the included studies in this meta-analysis. Maternal age at randomisation was similar between studies, with an average age of 30 years. BMI ranged between 24 and 35 kg/m^2^, with most studies including women with an average BMI of approximately 30 kg/m^2^. The majority of studies randomised women between 20 and 36 weeks of gestation [31,32,34,42,43,44,48,49,50,51,52,53,54,58], although others [36–39,55] randomised women between 11 and 33 weeks of gestation. Gestational age at study entry was consistent across studies, at around 30 weeks of gestation.

**Table 2:** Demographic information for women included in the meta-analysis.

First author	Country	Gestational age (weeks) at randomisation	Maternal age (years) at randomisation	BMI (kg/m^2^) at randomisation	Gestational age at entry (weeks)
Arshad (2017)	Egypt	12–24	M: 29.8 ± 3.4; I: 31.6 ± 4.3	Not recorded	Not recorded
Ashoush (2016)	Pakistan	22–36	M: 31.6 ± 2.8; I: 32.1 ± 3.2	M: 31.3 ± 1.3; I: 31.4 ± 1.5	M: 29.8 ± 1.4; I: 29.7 ± 1.9
Barrett (2013)	Australia	22–33	M: 33.3 (32.6–34); I: 32.9 (32.2–33.5)	M: 34.5 (33.5–35.5); I: 33.8 (32.8–34.8)	Not recorded
Battin (2015)	Australia	22–33	Not recorded	Not recorded	Not recorded
Bertini (2005)	Brazil	11–33	G: 31.2 ± 4.5; I: 28.7 ± 6.0	G: 27.5 ± 5.8; I: 27. ± 7.2	Not recorded
Borg (2016)	Egypt	22–36	M: 25 ± 4.6; I: 30 ± 4.0	M: 22.5 ± 4.9; I: 23.5 ± 3.7	Not recorded
Hassan (2012)	Pakistan	20–36	M: 30.3 ± 3.0; I: 30.9 ± 3.6	M: 29.2 ± 1.9; I: 28.7 ± 2.7	M: 29.5 ± 1.3; I: 29.2 ± 1.5
Ijas (2011)	Finland	12–34	M: 32.3 ± 5.6; I: 31.7 ± 5.6	M: 31.5 ± 6.5; I: 30.8 ± 5.4	Not recorded
Ijas (2015)	Finland	12–34	M: 32.1 ± 5.1; I: 31.9 ± 6.2	M: 31 ± 6.2; I: 30.6 ± 5.4	M: 30 ± 4.5; I: 30.4 ± 4.1
Khan (2017)	Pakistan	Not recorded	M: 24.9 ± 2.6; I: 28 ± 2.5	M: 22 ± 3; I: 23.8 ± 2.8	Not recorded
Lain (2009)	US	24–33	G: 32.2 ± 5.0; I: 31.2 ± 5.9	G: 33.4 ± 12.9; I: 30.9 ± 5.7	Not recorded
Langer (2000)	US	11–33	G: 29.0 ± 7.0; I: 30.0 ±6.0	Not recorded	G: 24.0 ± 7.0; I: 25.0 ± 7.0
Mirzamoradi (2013)	Iran	24–36	G: 29.5 ± 4.1; I: 31.2 ± 5.0	Not recorded	Not recorded
Moore (2007)	Mexico	20–34	Not recorded	Not recorded	Not recorded
Moore (2010)	Mexico	11–33	M: 31 ± 7.1; G: 29.6 ± 7.8	M: 32.8 ± 5.8; G: 32.7 ± 7.0	M: 27.3 ± 7; G: 29.1 ± 5
Mukhopadhyay (2012)	India	20–28	G: 26.3 ± 4.6; I: 26.0 ± 4.3	G: 23.7 ± 2.7; I: 23.0 ± 2.9	G: 28.3 ± 2.2; I: 27.4 ± 2.7
Niromanesh (2012)	Iran	20–34	M: 30.7 ± 5.5; I: 31.8 ± 5.1	M: 28.1 ± 4.0; I: 27.1 ± 2.1	M: 28.7 ± 3.7; I: 28.6 ± 3.6
Rowan (2008)	Australia	22–33	M: 33.5 ± 5.4; I: 33.0 ± 5.1	M: 35.1 ± 8.3; I: 34.6 ± 7.2	M: 30.2 ± 3.3; I: 30.1 ± 3.2
Rowan (2011)	Australia	22–33	M: 39.4 ± 5.4; I: 38.9 ± 5.0	M: 33.4 ± 12; I: 31.6 ± 10.0	M: 30.4 ± 3.3; I: 30.0 ± 3.3
Rowan (2018)	Australia	22–33	Not recorded	Not recorded	Not recorded
Saleh (2016)	Egypt	26–34	M: 31.0 ± 3.4; I: 29.8 ± 2.2	M: 30.5 ± 3.1; I: 31.6 ± 3.1	M: 27.3 ± 3.5; I: 29.3 ± 3.1
Senat(2018)	France	24–34	G: 32.5 ± 5.1; I: 32.6 ± 5.3	G: 30.7 ± 5.1; I: 31.1 ± 5.4	Not recorded
Silva (2007)	Brazil	11–33	G: 31.6 ± 4.2; I: 29.9 ± 6.0	Not recorded	Not recorded
Silva (2010)	Brazil	11–33	M: 33.6 ± 5.8; G: 31.5 ± 5.4	Not recorded	M: 26.8 ± 6; G: 25.6 ± 6.4
Silva (2012)	Brazil	11–33	M: 31.3 ± 5.4; G: 32.6 ± 5.6	M: 28.7 ± 5.4; G: 28.6 ± 5.9	M: 25.4 ± 7; G: 26.9 ± 6.4
Somani (2016)	India	24–34	M: 25.6 ± 4.7; I: 26.3 ± 3.8	Not recorded	M: 27.8 ± 2.5; I: 28.3 ± 2.6
Spaulonci (2013)	Brazil	24–28	M: 31.9 ± 6.0; I: 32.8 ± 4.7	M: 32.0 ± 4.8; I: 34.1 ± 5.7	M: 32.2 ±3.7; I: 32.0 ± 3.5
Tempe (2013)	India	22–34	Not recorded	Not recorded	Not recorded
Tertti (2013)	Finland	22–34	M: 31.5 ± 5.0; I: 32.1 ± 5.4	M: 29.4 ± 5.9; I: 28.9 ± 4.7	M: 30.3 ± 2; I: 30.4 ± 2
Tertti (2014)	Finland	22–34	Not recorded	Not recorded	Not recorded
Tertti (2015)	Finland	22–34	Not recorded	M: 32.3 ± 5.2; I: 31.7 ± 5.0	Not recorded
Tertti (2016)	Finland	22–34	Not recorded	Not recorded	Not recorded
Wouldes (2016)	Australia	22–33	Not recorded	Not recorded	Not recorded

All outcomes signified with plus-minus sign ‘±’ are SD; outcomes signified with parentheses ‘()’ are 95% CI.

Abbreviations: CI, confidence interval; G, glyburide; I, insulin; M, metformin

Measures of maternal glycaemic control at the end of pregnancy were not significantly different for any of the treatment comparisons when assessed by FBS, RBS, or HbA1c (S5 Fig). In all included studies, supplementation with insulin was available to achieve glycaemic control within target ranges. The treatment failure rate (the number of women who needed supplementation with insulin to maintain glycaemic control) in the glyburide versus insulin comparisons ranged from 0% to 21%, with a weighted average failure rate of 12.9%. In metformin versus insulin comparisons, treatment failure rates ranged from 14% to 46%, with a weighted average failure rate of 33.2%. In the studies directly comparing metformin versus glyburide, women were more likely to require supplementary insulin when treated with metformin than with glyburide (OR 0.62, 95% confidence interval [CI] 0.40–0.97, I_2_ = 45%, *p* = 0.04).

**Fig 2:**
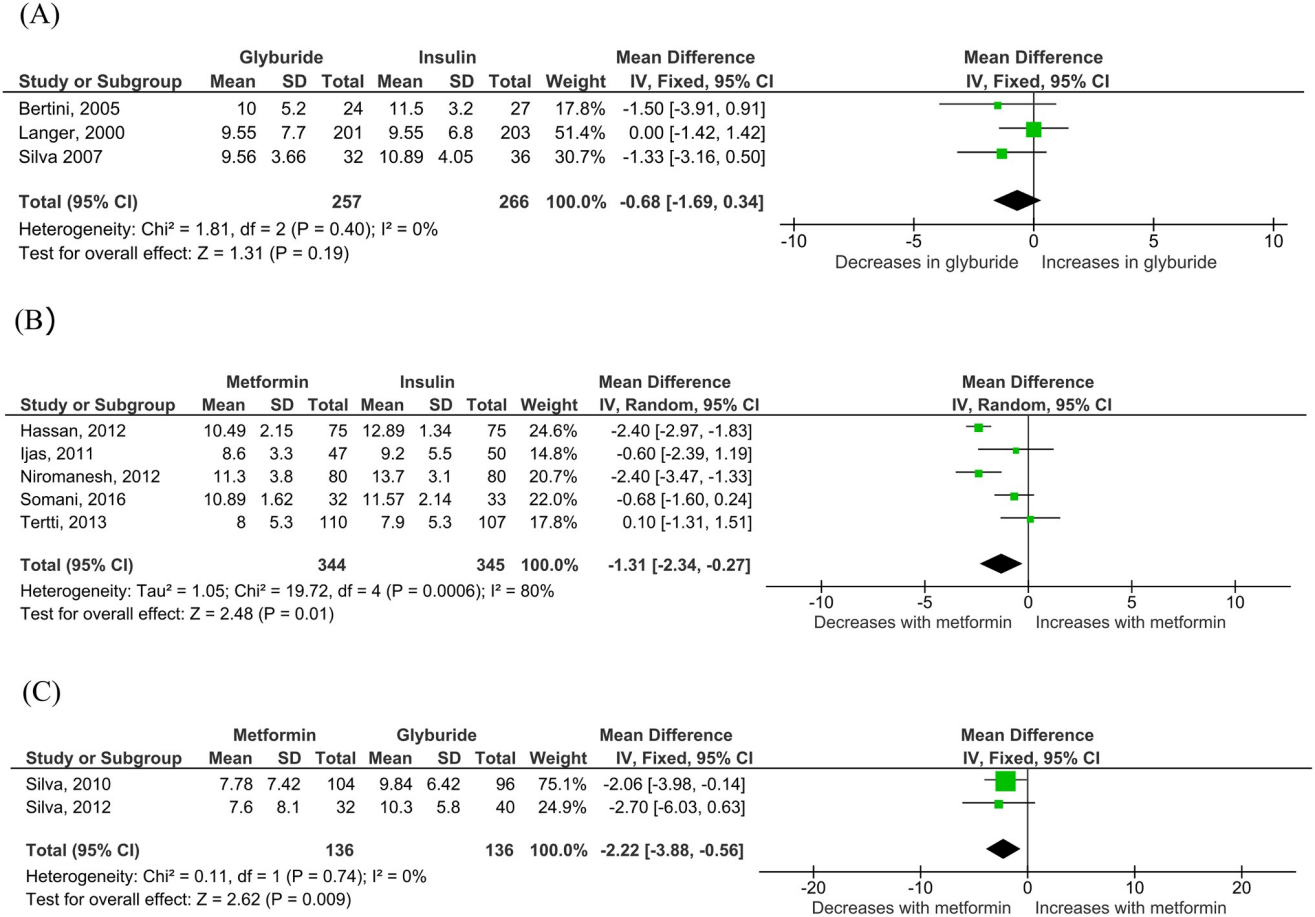
Total gestational weight gain for (A) glyburide versus insulin, (B) metformin versus insulin, and (C) metformin versus glyburide comparisons. Expressed as mean difference and fixed effects model, except metformin versus insulin (expressed as random effects model). All comparisons 95% CI. CI, confidence interval.

Three studies [35,36,53], including 523 women, recorded total gestational weight gain in glyburide compared to insulin-treated women. No difference in total gestational weight gain was observed between glyburide versus insulin-treated women (−0.68 kg; 95% CI −1.69 kg to 0.34 kg; I_2_ = 0%, *p* = 0.19) (Fig 2A). Five studies [33,34,40,42,56], including 689 women, measured gestational weight gain in women treated with metformin versus insulin. Metformin-treated mothers gained less weight over the total pregnancy compared to those treated with insulin (−1.31 kg; 95% CI −2.34 kg to −0.27 kg; I_2_ = 80%, *p* = 0.01) (Fig 2B). Two studies [49, 50], including 272 women, showed that those treated with metformin also gained significantly less weight over the whole pregnancy compared to those treated with glyburide (−2.20 kg; 95% CI −3.88 kg to −0.56 kg; I_2_ = 0%, *p* = 0.009) (Fig 2C). Six studies [31,34,43,40,41,44], including 1,295 women, reported posttreatment weight gain in metformin- versus insulin-treated women. This was very similar to the magnitude of difference in total gestational weight gain (−1.31kg), demonstrating that the difference in weight gain between these groups was likely due primarily to the allocated treatment (−1.15 kg; 95% CI −1.87 kg to −0.42 kg, I_2_ = 96%, *p* = 0.002). No studies comparing glyburide to either insulin or metformin reported this outcome measure.

With respect to maternal outcomes, several analyses failed the LOO sensitivity analysis (gestational weight gain comparing metformin with insulin, gestational weight gain comparing metformin with glyburide, and FBS comparing metformin with insulin), and these analyses should therefore be interpreted with caution.

### Fetal and neonatal outcomes

**Fig 3:**
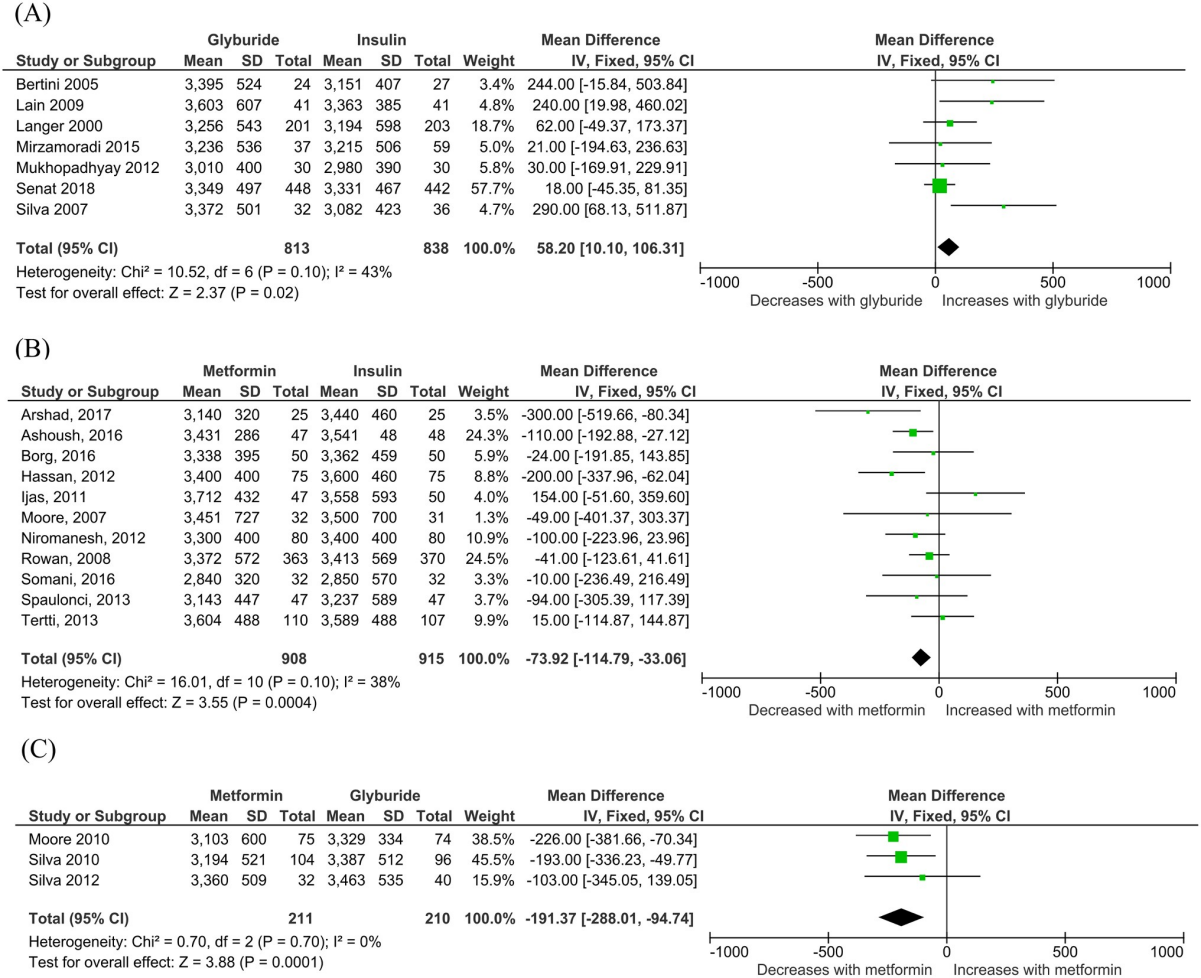
Birth weight for (A) glyburide versus insulin, (B) metformin versus insulin, and (C) metformin versus glyburide comparisons. Expressed as mean difference (fixed effects model) and 95% CI. CI, confidence interval.

No eligible studies in any of the treatment comparison groups (glyburide versus insulin, metformin versus insulin, or metformin versus glyburide) reported any fetal growth data. In seven studies including 1,651 participants [32,35,36,37,50,53,58], neonates exposed to glyburide were significantly heavier at birth (58.20 g; 95% CI 10.10 g to 106.31g; I_2_ = 43%, *p* = 0.02) compared to those born to mothers treated with insulin (Fig 3A). In 11 studies including 1,823 women [30,31,33,34,40–44,54,56], metformin-exposed neonates were significantly lighter at birth compared to insulin-exposed neonates, with average birth weights 73.92 g lighter (95% CI −114.79 g to −33.06 g, I_2_ = 38%, *p* < 0.001) (Fig 3B). In three studies [38,39,55] including 421 participants, metformin-exposed neonates were lighter by an average of 191.37 g (95% CI −288.01 g to −94.74 g, I_2_ = 0%, *p* < 0.001) compared to those exposed to glyburide (Fig 3C).

**Fig 4:**
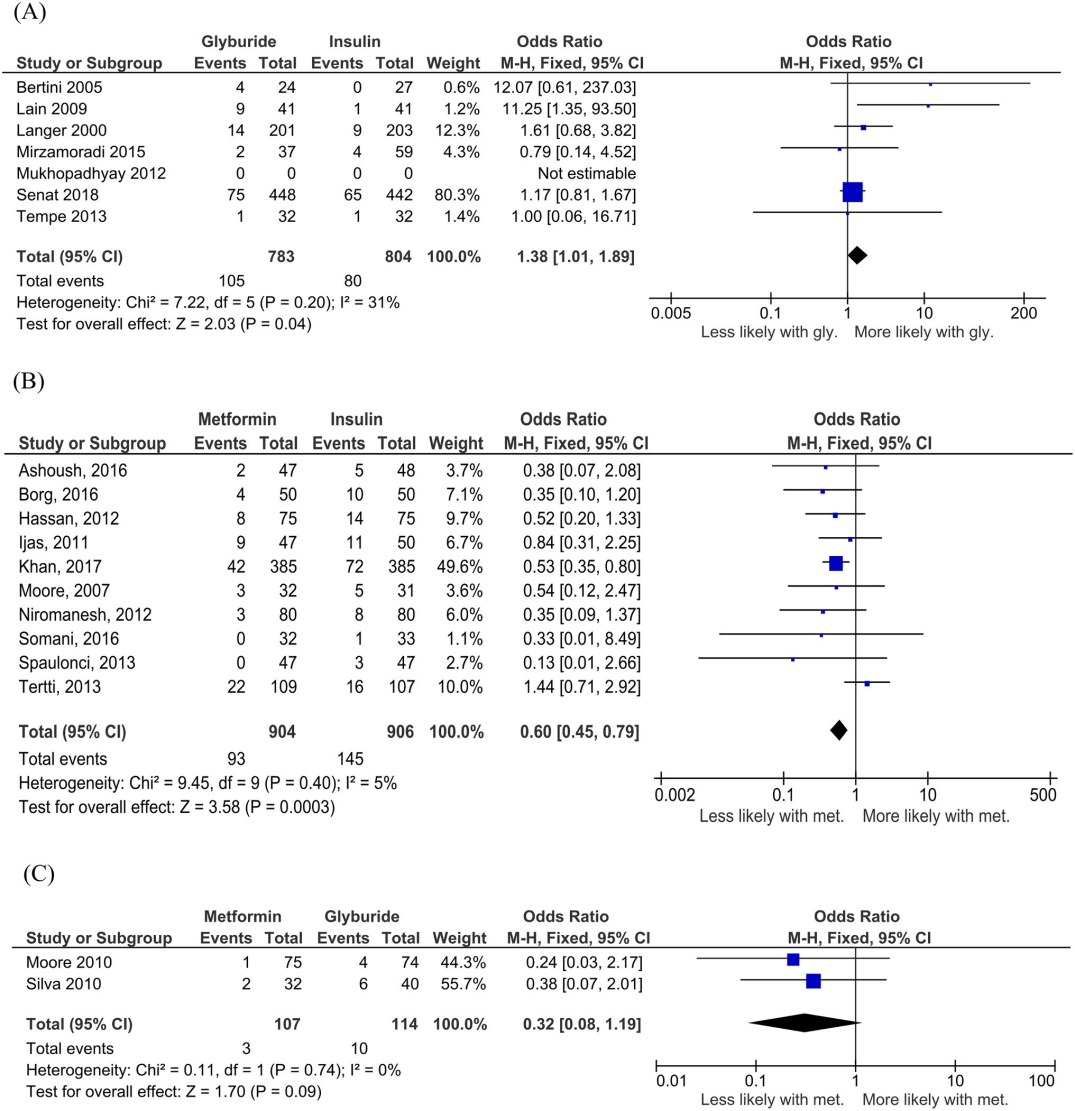
Macrosomia for (A) glyburide versus insulin, (B) metformin versus insulin, and (C) metformin versus glyburide comparisons. Expressed as mean difference (random effects model). Expressed as OR (fixed effects model) and 95% CI. CI, confidence interval; OR, odds ratio.

In seven studies including 1,587 participants [32,35,36,50,53,58,59], neonates exposed to glyburide had increased rates of macrosomia compared to insulin-exposed neonates (OR 1.38, 95% CI 1.01 to 1.89; I_2_ = 31%, *p* = 0.04) (Fig 4A). In 10 studies including 1,810 women [31,33,34,40,41,42,44,45,54,56], metformin-exposed neonates had reduced rates of macrosomia compared to insulin-exposed neonates (OR 0.60, 95% CI 0.45 to 0.79, I_2_ = 5%, *p* < 0.001) (Fig 4B). In two studies including 221 women [38,55], there was a nonsignificant decrease in macrosomia in metformin-exposed compared to glyburide-exposed neonates, although this did not reach statistical significance (OR 0.32, 95% CI 0.08 to 1.19, I_2_ = 0%, *p* = 0.09) (Fig 4C).

**Fig 5:**
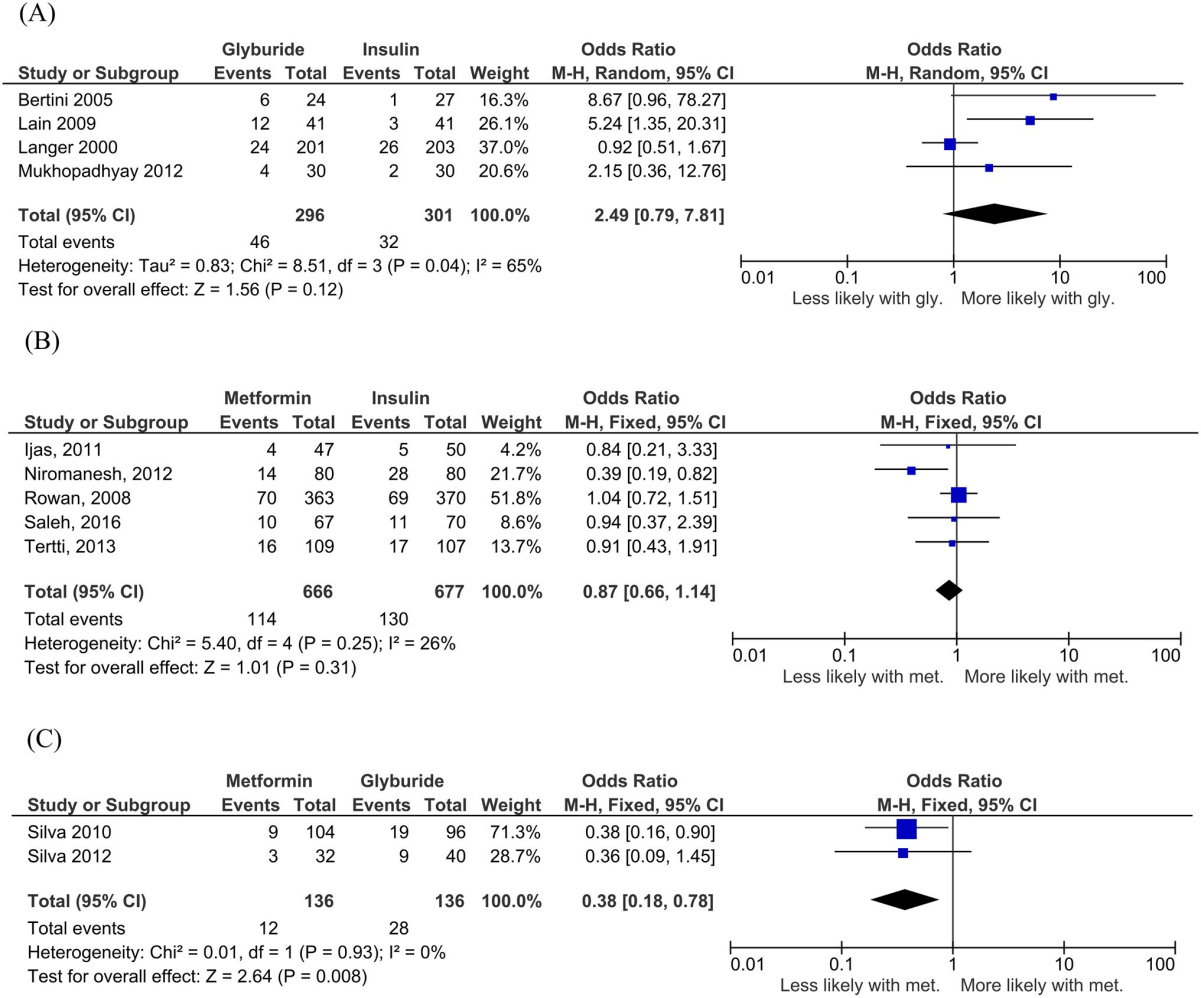
LGA for (A) glyburide versus insulin, (B) metformin versus insulin, and (C) metformin versus glyburide comparisons. Expressed as OR (fixed effects model) with the exception of the glyburide versus insulin comparison (random effect model). All comparisons 95% CI. CI, confidence interval; LGA, large for gestational age; OR, odds ratio.

In meta-analyses comparing LGA rates between groups, although the trends were similar to meta-analyses for macrosomia, there were fewer studies available for analysis. In four studies including 587 women [35,36,53,58], LGA rates did not show a statistically significant difference between glyburide-exposed versus insulin-exposed neonates (OR 2.49, 95% CI 0.79 to 7.81, I_2_ = 65%, *p* = 0.12) (Fig 5A). In five studies including 1,343 women [33,34,40,43,45], LGA incidence was unchanged between metformin- and insulin-exposed neonates (OR 0.87, 95% CI 0.66 to 1.14, I_2_ = 26%, *p* = 0.31) (Fig 5B). In two studies of 272 women [38,39], LGA was significantly decreased in metformin-exposed neonates compared to those exposed to glyburide in utero (OR 0.38, 95% CI 0.18 to 0.78, I_2_ = 0%, *p* = 0.008) (Fig 5C).

Neonatal ponderal index was reported in two studies [50,53], including 974 women in glyburide versus insulin comparisons. Glyburide-exposed neonates had a similar ponderal index compared to insulin-exposed neonates (−0.01, 95% CI −0.49 to 0.46, I_2_ = 0%, *p* = 0.96). In metformin versus insulin comparisons, three studies of 986 women [41,43,47] demonstrated that neonates exposed to metformin had a reduced ponderal index compared to insulin-exposed neonates (−0.13, 95% CI −0.26 to −0.00, I_2_ = 0%, *p* = 0.04). Only one study [39] including 200 women reported ponderal index comparing glyburide with metformin. Neonates exposed to metformin during gestation had decreased ponderal index (−0.09, 95% CI −0.17 to −0.01, I_2_ = non-applicable [N/A], *p* = 0.03) compared to neonates exposed to glyburide (Table 3).

**Table 3:** Neonatal anthropomorphic characteristics.

Anthropomorphic characteristics	Glyburide versus insulin	Metformin versus insulin	Metformin versus glyburide
Ponderal index	−0.01 [−0.49 to 0.46]	−0.13 [−0.26 to −0.00] ↓	−0.09 [−0.17 to −0.01] ↓
Head circumference	0.30 [−0.31 to 0.91]	−0.21 [−0.39 to −0.03] ↓	*No studies*
Chest circumference	0.80 [0.07 to 1.53] ↑	−0.34 [−0.62 to −0.05] ↓	*No studies*
Abdominal circumference	0.90 [0.03 to 1.77] ↑	0.00 [−0.44 to 0.44]	*No studies*
Total fat mass	102.3 [−3.91 to 210.91]^†^	*No studies*	*No studies*
Triceps skinfold thickness	0.00 [−0.35 to 0.35]	0.10 [−0.11 to 0.31]	*No studies*
Subscapular skinfold thickness	0.40 [−0.10 to 0.90]	0.00 [−0.20 to 0.20]	*No studies*

All outcomes expressed as mean difference. All outcomes used fixed model effects and 95% CI. Units: head circumference, cm; chest circumference, cm; abdominal circumference, cm; ponderal index, kg/m^3^.

^†^Increase not meeting statistical significance (*p* = 0.06).

^↑^ Increased (*p* < 0.05).

^↓^ decreased (*p* < 0.05).

Only one study of 82 women [53] reported neonatal anthropometric outcomes (neonatal head, chest, and abdominal circumferences) in glyburide versus insulin comparisons. Neonates exposed to glyburide during gestation had similar head circumferences (0.30 cm, 95% CI −0.31 to 0.91, I_2_ = N/A, *p* = 0.30) and increased chest (0.80 cm, 95% CI 0.07–1.53, I_2_ = N/A, *p* = 0.02) and abdominal circumferences (0.90 cm, 95% CI 0.03–1.77, I_2_ = N/A, *p* = 0.04) compared to insulin-exposed babies. Neonatal anthropometry was reported in up to three studies including 986 women that compared metformin to insulin [33,41,43]. Metformin-exposed babies had smaller head (−0.21 cm, 95% CI −0.39 to −0.03, I_2_ = 53%, *p* = 0.02) and chest circumferences (−0.34 cm, 95% CI −0.62 to −0.05, I_2_ = 42%, *p* = 0.02), yet no difference in abdominal circumference (0.00 cm, 95% CI −0.44 cm to 0.44 cm, I_2_ = N/A, *p* = 1.00), compared to those exposed to insulin (Table 3). In metformin versus glyburide studies, no neonatal anthropometry was reported.

Only one study [53] of 71 women compared glyburide with insulin and reported measures of neonatal adiposity. This demonstrated a nonsignificant increase in total fat mass in glyburide- versus insulin-exposed neonates (102.3 g, 95% CI −3.91 g to 210.91 g, I_2_ = N/A, *p* = 0.06). Tricep skinfold (0.10 cm, 95% CI, −0.11 cm to 0.31 cm, I_2_ = N/A, *p* = 0.34) or subscapular skinfold thicknesses were unchanged (0.00 cm, 95% CI −0.35 cm to 0.35 cm, I_2_ = N/A, *p* = 1.00) between glyburide- and insulin-exposed groups. In metformin versus insulin comparisons, one study of 733 women [43] reported neonatal adiposity indices. This study demonstrated no difference in neonatal tricep skinfold (0.10 cm, 95% CI −0.11 cm to 0.31 cm, I_2_ = N/A, *p* = 0.34) or subscapular skinfold thicknesses (0.00 cm, 95% CI −0.20 cm to 0.20 cm, I_2_ = N/A, *p* = 1.00); however, neonatal total fat mass was not reported. No fat anthropometric data were reported for the metformin versus glyburide comparison (Table 3).

With respect to neonatal outcomes, several analyses failed LOO sensitivity analysis (birth weight comparing glyburide with insulin, LGA comparing metformin with glyburide, and macrosomia comparing metformin with insulin), and these analyses should therefore be interpreted with caution.

### Later postnatal outcomes

No eligible studies comparing glyburide with insulin or with metformin looked at any postnatal growth outcomes later than the neonatal period. Previously, we reported that studies comparing metformin with insulin have shown increased infant weight, increased childhood BMI, and evidence of increased adiposity [29].

## Discussion

Our results demonstrate that babies exposed to glyburide are significantly heavier at birth and have increased incidence of macrosomia compared to those whose mothers were randomised to insulin. Conversely, metformin-exposed babies are significantly lighter at birth and have reduced incidence of macrosomia compared to insulin-exposed babies. Babies whose mothers were randomised to metformin versus glyburide were significantly lighter and significantly less likely to be born LGA. In addition to birth weight differences, our findings also suggest that there may be differences in neonatal body composition between treatment groups. In particular, glyburide-exposed babies have increased abdominal circumference and a nonsignificant increase in total fat mass compared to the insulin-exposed group, and higher ponderal index compared to the metformin-exposed group. Conversely, babies born to mothers treated with metformin have a reduced ponderal index compared to either insulin- or glyburide-exposed babies. Although limited suitable studies are available for inclusion in these meta-analyses, a clear picture emerges of babies exposed to glyburide being born larger and with increased adipose mass, and babies exposed to metformin being born smaller and with reduced lean mass, compared to those treated with standard insulin therapy.

Despite the significant differences in neonatal anthropometry at birth, we found that metformin, glyburide, and insulin were all equally effective for maternal glycaemic control (when supplementary insulin was available as required), as evidenced by FBS, RBS, or HbA1c measurement. Although there are there are limitations associated with the use of HbA1c as an index of glycaemic control in GDM (as it reflects glycaemic control over several months), the observation that all three different assessments led to the same conclusion gives confidence in this finding. Moreover, no study utilised HbA1c to assess glycaemic control alone. With regard to the relative merits of treating GDM with metformin versus glyburide from a maternal point of view, we demonstrate that metformin has a higher likelihood of treatment failure and requiring supplementary insulin than glyburide, but it is also associated with less gestational weight gain than either glyburide or insulin.

### Strengths

Our study design incorporates all key comparisons required to fully evaluate the impact of pharmacological treatment of GDM on neonatal anthropometry and maternal glycaemic control. The ability to perform the numerous relevant comparisons within a single comprehensive study design is a major strength of this work. Having fully recognised the paucity of studies reporting key data for some outcomes, we have performed extensive evaluation of the robustness of our conclusions, and urge a conservative view to interpretation. However, even allowing for this highly cautious approach, a clear pattern of nonequivalence amongst therapies emerges with respect to neonatal size and body composition. Where possible, we have performed multiple sensitivity analyses to take account of other variables, for example, the differing criteria used to diagnose GDM and the various global contexts within which the studies were performed.

### Limitations

The ability to draw definitive conclusions from our meta-analysis is limited by both the quantity and quality of the studies available. With respect to glyburide, in particular, few studies met the inclusion criteria (8 studies comparing glyburide to insulin, versus 22 studies comparing metformin to insulin). Moreover, our comparisons of metformin versus glyburide treatment are based on relatively sparse data relating to only 421 women (3 studies). Our findings highlight the surprising lack of high-quality data on which to base clinical recommendations regarding oral anti-hyperglycaemic agents in the treatment of GDM. The lack of high-quality data results in the need for caution in interpretation of the analyses that are not robust to sensitivity testing.

None of the eligible studies reported the effects of pharmacological intervention for GDM on fetal growth outcomes. Clearly, weight and body composition at birth are products of fetal growth in utero, and without fetal data, it is challenging to deduce what underlying mechanisms may be driving the observed neonatal outcomes. In light of our findings, there is an urgent need to report fetal growth data in future studies. Similarly, we were unable to compare postnatal growth patterns between treatment groups. No eligible studies comparing glyburide to either insulin or metformin have reported any growth outcomes beyond the immediate neonatal phase. We have previously reported that children exposed to metformin versus insulin in utero are smaller at birth but have accelerated postnatal growth [29]. Our findings of altered birth weight and fat mass in babies exposed to oral anti-hyperglycaemic drugs in utero highlight a future research need for longitudinal studies of growth and body composition following intrauterine glyburide or metformin exposure.

### Interpretation and implications

When GDM is managed using diet and lifestyle interventions, or with insulin, maternal glycaemic control can be used as a proxy for fetal growth outcomes [3]. Although insulin does not cross the placental barrier [60,61], metformin and glyburide both do. Our results imply that other direct and indirect mechanisms impact on regulation of fetal growth when oral anti-hyperglycaemic agents are used in GDM treatment.

Glyburide has been detected in umbilical cord blood concentrations up to 70% of those observed in the maternal circulation [25–27]. Glyburide is thought to be transported across the placenta via ATP-binding cassette protein (ABC) transporters located in both the apical and basolateral membranes of the syncytiotrophoblast. The relationship between maternal and fetal glyburide levels is complicated by the drug’s high protein binding and active feto-maternal efflux [25,62]. The normalisation of maternal euglycaemia by glyburide relies on stimulating increased insulin production from maternal beta cells. However, as glyburide also reaches the fetal beta cells across the placental barrier, these may also be directly stimulated to excess insulin production. Fetal plasma insulin is a critical regulator of fetal growth, linking both nutrient supply and anabolic signals for growth [63]. There is therefore a plausible mechanism by which direct fetal exposure to glyburide and stimulation of fetal insulin secretion could result in fetal overgrowth and adiposity, even in the absence of maternal hyperglycaemia.

**Fig 6:**
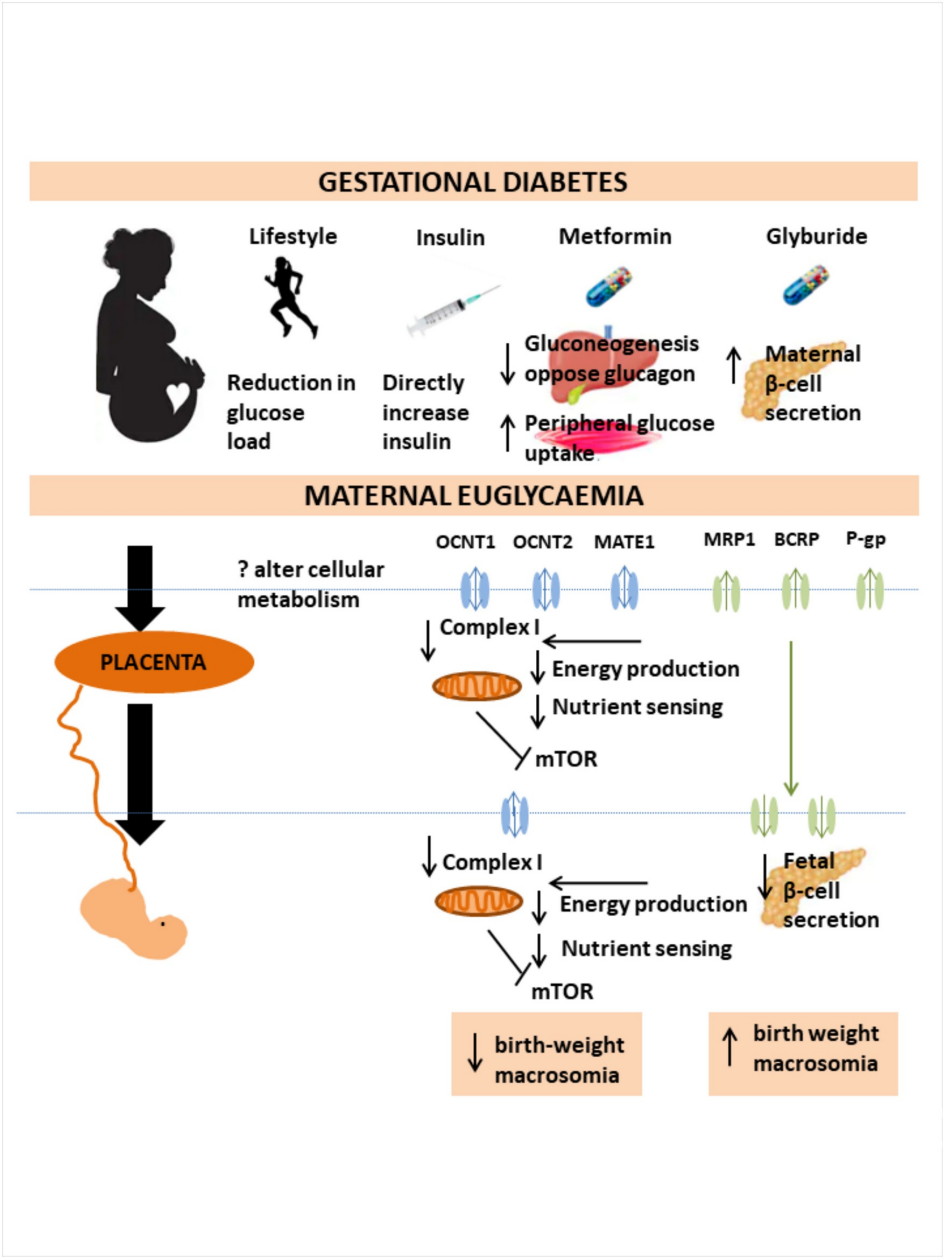
Summary diagram of the mechanisms of action of GDM intervention on mother and baby. GDM, gestational diabetes mellitus.

Fetal circulating metformin levels can approach or exceed those in the maternal circulation [22–24]. Organic cation transporters, key to transporting metformin intracellularly, are present in both placental and fetal tissues by the time GDM treatment is initiated in the second and third trimester [64,65]. One of metformin’s major intracellular effects is on the mammalian target of rapamycin (mTOR) pathway, known to be important for nutrient sensing, by both 5′ AMP-activated protein kinase (AMPK)-dependent [66] and AMPK-independent [67] mechanisms. It is possible that metformin-induced mTOR inhibition could restrict placental nutrient transfer, hence limiting transport of glucose and amino acids [68] and potentially providing a mechanism for lower birth weight and reduced ponderal index observed in metformin-exposed neonates compared to their insulin- or glyburide-exposed counterparts. Metformin reduces cellular energy production via inhibition of complex I of the mitochondrial electron transport chain, leading to an increase in both ADP/ATP and AMP/ATP ratios, inhibiting (mTOR) actions and down-regulating pathways associated with cell growth and proliferation [69,70]. There is therefore an urgent research need to investigate these potential mechanisms by which oral anti-hyperglycaemic agents could impact on fetal growth (Fig 6), particularly in view of their increasing clinical use.

Our results suggest that there may not be a single optimal pharmacological agent for treatment of GDM across all global contexts. Birth weight was increased by glyburide (versus insulin) to a greater extent in studies performed in the US/Latin America than in other global contexts, whereas studies from North Africa/Middle East showed a greater decrease in birth weight with metformin (versus insulin) than studies performed elsewhere. This potentially reflects different underlying mechanisms of gestational diabetes in different populations, for example, peripheral insulin resistance versus beta cell dysfunction, potentially due to differing genetic backgrounds or different environmental challenges. There may also be population differences in patient preference for treatment options (for example, insulin may be preferred to adding a second oral agent [71]), which are important to consider given that outcomes rely on treatment concordance. In addition to the logistic challenges of delivering a context-appropriate agent (for example, avoiding insulin where refrigeration is not practicable), our results suggest that the efficacy of anti-hyperglycaemic agents in promoting normal fetal growth may vary across contexts. This is an important target for future research to inform health policy and reflects heterogeneity in the condition of GDM.

Our findings show significant differences in neonatal body weight and anthropometric parameters between babies whose mothers were randomised to glyburide, metformin, and insulin to treat GDM, even with equivalent maternal glycaemic control. Our results highlight the importance of considering the effects of treatment on both mother (glycaemic control) and baby (fetal growth) when managing GDM. The lack of data for inclusion in these meta-analyses is concerning, given that numerous bodies worldwide already endorse the use of oral anti-hyperglycaemic agents as first- or second-line treatments for GDM [14,15,17,18]. Examining in detail the mechanisms by which the various pharmacological options for GDM treatment may impact the developing fetus and/or placenta in both the short and long term is particularly important, given the increasing incidence of GDM worldwide [1,2]. Most significantly, the greatest increases in GDM globally are occurring in populations in which treating GDM with insulin is unlikely to be feasible for large numbers of women [72]. Further understanding of how oral anti-hyperglycaemic agents impact on fetal growth trajectory and later life outcomes should therefore be a research priority. There is an urgent need for further data to define the potential risk associated with GDM treatments and lifetime risk of adverse metabolic outcomes for the infant.

## Supporting information

S1 PRISMA Checklist PRISMA, Preferred Reporting Items for Systematic Reviews and Meta-Analyses. (DOC)

S1 Text PROSPERO protocol. CRD42019134664.
(PPTX)

S2 Text Paper databases search strategies. (A) PubMed, (B) OVID EMBASE, (C) Medline, (D) Web of Science, (E) Cochrane Library, and (F) www.clinicaltrials.gov.
(PPTX)

S1 Table Inclusion/exclusion critieria table. (PPTX)

S2 Table Risk of bias assessment. (A) Random sequence generation (selection bias), (B) allocation concealment (selection bias), (C) blinding participants and personnel (performance bias), (D) blinding of outcome assessment (detection bias), (E) incomplete outcome data (attrition bias), (F) selection bias (reporting bias), and (G) other bias.
(PPTX)

S3 Table Study characteristics table. (XLSX)

S4 Table Egger’s test table. (PPTX)

S1 Fig Leave-one-criteria-out sensitivity analysis for birth weight for (A) glyburide versus insulin, (B) metformin versus insulin, and (C) metformin versus glyburide. ADA, ADIPS, BHM, CC, FNC, IADPSG, NDDG, WHO, and no criteria detailed. ADA, American Diabetes Association; ADIPS, Australasian Diabetes in Pregnancy Society; BHM, Brazilian Health Ministry; CC, Carpenter-Coustan; FNC, Finnish National Criteria; IADPSG, International Association of Diabetes and Pregnancy Study Groups; NDDG, National Diabetes Data group; WHO, World Health Organization.
(PPTX)

S2 Fig Leave-one-continent-out sensitivity analysis for birth weight for (A) glyburide versus insulin and (B) metformin versus insulin. (PPTX)

S3 Fig LOO sensitivity analysis. (A) Gestational weight gain, (B) birth weight, (C) macrosomia, (D) LGA, (E) FBS, (F) RBS, and (G) HbA1c in glyburide versus insulin, metformin versus insulin, and metformin versus glyburide comparisons. All outcomes expressed as OR (95% CI). CI, confidence interval; FBS, fasting blood glucose; HbA1c, glycated haemoglobin; LGA, large for gestational age; LOO, leave-one-out; OR, odds ratio; RBS, random blood glucose.
(PPTX)

S4 Fig Funnel plots to assess publication bias. All outcomes plotted.
(PPTX)

S5 Fig Maternal glycaemic control: (A) FBS, (B) RBS, and (C) HbA1c in glyburide versus insulin, metformin versus insulin, and metformin versus glyburide comparisons. All outcomes and comparisons were N/S. FBS, fasting blood glucose; HbA1c, glycated haemoglobin; RBS, random blood glucose.
(PPTX)

## Abbreviations

ABC: ATP-binding cassette protein
ACOG: American College of Obstetrians and Gynecologists
ADA: American Diabetes Association
ADIPS: Australasian Diabetes in Pregnancy Society
AMPK: 5′ AMP-activated protein kinase
BHM: Brazilian Health Ministry
CC: Carpenter-Coustan
CDA: Canadian Diabetes Association
CI: confidence interval
FBS: fasting blood glucose
FDA: Food and Drug Administration
FNC: Finnish National Criteria
GDM: gestational diabetes mellitus
HbA1c: glycated haemoglobin
IADPSG: International Association of Diabetes and Pregnancy
ITT: intention-to-treat
LGA: large for gestational age
LOO: leave-one-out
mTOR: mammalian target of rapamycin
N/A: Non-Applicable
NDDG: National Diabetes Data Group
NICE: National Institute for Clinical Excellence
OR: odds ratio
PRISMA: Preferred Reporting Items for Systematic Reviews and Meta-Analyses
RACGP: Royal Australian College of General Practitioners
RBS: random blood glucose
SMFM: Society of Maternal-Fetal Medicine
WHO: World Health Organization
