# Supplementary material for: Comparative impact of pharmacological treatments for gestational diabetes on neonatal anthropometry independent of maternal glycaemic control: A systematic review and meta-analysis
Source: PLoS Med. 2020 May 22;17(5):e1003126. doi: 10.1371/journal.pmed.1003126 (PMC7244100; doi:10.1371/journal.pmed.1003126)
Supplement: S1 PRISMA Checklist — (DOC) [file pmed.1003126.s001.doc]

| **Section/topic** | **#** | **Checklist item** | **Reported on page #** |
| --- | --- | --- | --- |
| **TITLE** | | |  |
| Title | 1 | Identify the report as a systematic review, meta-analysis, or both. “Comparative impact of pharmacological treatments for gestational diabetes on neonatal anthropometry independent of maternal glycaemic control: a systematic review and meta-analysis.” | Title, paragraph 1 |
| **ABSTRACT** | | |  |
| Structured summary | 2 | Provide a structured summary including, as applicable: background; objectives; data sources; study eligibility criteria, participants, and interventions; study appraisal and synthesis methods; results; limitations; conclusions and implications of key findings; systematic review registration number. “***Background*** Regulation of fetal growth in gestational diabetes mellitus (GDM) is directly linked to maternal glycaemic control, however this relationship maybe altered by oral anti-hyperglycaemic agents. These drugs cross the placenta and may thus have independent effects on fetal or placental tissues. We aimed to investigate the association between GDM treatment and fetal, neonatal and childhood-growth. ***Methods and findings*** PubMed, Ovid Embase, Medline, Web of Science, ClinicalTrials.gov, and Cochrane databases were systematically searched (from database…)” | Abstract, paragraphs 7-9. |
| **INTRODUCTION** | | |  |
| Rationale | 3 | Describe the rationale for the review in the context of what is already known. “Gestational diabetes mellitus (GDM) is an increasing healthcare concern, affecting 3 to 25% of pregnancies worldwide [1]. It was estimated in 2017 that approximately one in seven babies globally was born to a mother with GDM [2]. If untreated, GDM has significant short and long-term health implications for mother and baby, making optimal clinical management of GDM an important priority. Poorly managed or untreated GDM leads to accelerated fetal …” | Introduction, paragraph 11. |
| Objectives | 4 | Provide an explicit statement of questions being addressed with reference to participants, interventions, comparisons, outcomes, and study design (PICOS). “The aim of this study is to provide a systematic, unbiased, and comprehensive overview of the comparative impacts of various pharmacological treatments for GDM on fetal growth, neonatal anthropometry, and childhood growth outcomes, including considering the effects of maternal glycaemic control.” | Introduction, paragraph 14. |
| **METHODS** | | |  |
| Protocol and registration | 5 | Indicate if a review protocol exists, if and where it can be accessed (e.g., Web address), and, if available, provide registration information including registration number. “This systematic review and meta-analysis was conducted in accordance with the Preferred Reporting Items for Systematic Reviews and Meta-Analyses (PRISMA) guidelines [28]. The PRISMA checklist is detailed in (S1 PRISMA checklist). The systematic review protocol was registered in PROSPERO CRD42019134664 (S1 Text). Metformin *vs.* insulin data was derived from PROSPERO protocol CRD42018117503 [30]. Ethical approval was not required”. | Methods and Materials, paragraph 15 and S1 Text. |
| Eligibility criteria | 6 | Specify study characteristics (e.g., PICOS, length of follow-up) and report characteristics (e.g., years considered, language, publication status) used as criteria for eligibility, giving rationale. “Studies that randomised women with GDM to glyburide *vs.* insulin therapy, metformin *vs.* insulin therapy, and metformin *vs.* glyburide therapy were included. Studies were excluded if they investigated other oral anti-glycaemic agents (such as myo-inositol), or if interventions were given prior to pregnancy. GDM was screened for and diagnosed according to local criteria in each study, and we did not apply exclusions with respect to this. Studies were excluded…” | Methods and Materials, (Literature searches, search strategies and eligibility criteria) Paragraph 16. |
| Information sources | 7 | Describe all information sources (e.g., databases with dates of coverage, contact with study authors to identify additional studies) in the search and date last searched. “Systematic literature searches using pre-specified terms (S2 text) were performed on PubMed (June 1997 to 7^th^ May 2019), Ovid EMBASE (1974 to 16^th^ September 2019), Ovid Medline (1946 to 16^th^ September 2019), Cochrane library (database inception to 16^th^ September 2019), Clinicaltrials.gov (database inception to 16^th^ September 2019), and Web of Science (1900 to 16^th^ September 2019). Details for the database search strategies for metformin *vs.* insulin comparisons can be found in [29]. An updated search (16^th^ September 2019) was also conducted to ensure no new papers were missed. No filters were applied to any of the searches.” | Methods and Materials (Literature searches, search strategies and eligibility criteria)  Paragraph 16. |
| Search | 8 | Present full electronic search strategy for at least one database, including any limits used, such that it could be repeated.  “(A) Search criteria for PubMed: ("glyburide"[MeSH Terms] OR "glyburide"[All Fields]) AND ("diabetes mellitus"[MeSH Terms] OR ("diabetes"[All Fields] AND "mellitus"[All Fields]) OR "diabetes mellitus"[All Fields]) AND ("pregnancy in diabetics"[MeSH Terms] OR ("pregnancy"[All Fields] AND "diabetics"[All Fields]) OR "pregnancy in diabetics"[All Fields] OR ("diabetes"[All Fields] AND "pregnancy"[All Fields]) OR "diabetes in pregnancy"[All Fields] OR "diabetes, gestational"[MeSH Terms] OR ("diabetes"[All Fields] AND "gestational"[All Fields]) OR "gestational diabetes"[All Fields] OR ("diabetes"[All Fields] AND "pregnancy"[All Fields]))” | S2 Text. |
| Study selection | 9 | State the process for selecting studies (i.e., screening, eligibility, included in systematic review, and, if applicable, included in the meta-analysis). “Two reviewers (JLA and CEA) independently assessed each study using pre-determined inclusion/exclusion criteria (detailed in S1 Table). A third reviewer (SEO) was available to resolve cases where eligibility was unclear. An initial screen of titles and abstracts was performed, followed by a detailed full paper screen (Fig 1). The results from each step of the review process are documented in a PRISMA flow diagram (Fig 1). | Methods and Materials (Study selection and data extraction). Paragraph 17. |
| Data collection process | 10 | Describe method of data extraction from reports (e.g., piloted forms, independently, in duplicate) and any processes for obtaining and confirming data from investigators. “Data extraction from eligible studies was conducted independently by two authors (JLA and CEA)”. “Where insufficient information for assessment was available, authors were contacted for further information.” | Methods and Materials (Study selection and data extraction). Paragraph 18. |
| Data items | 11 | List and define all variables for which data were sought (e.g., PICOS, funding sources) and any assumptions and simplifications made. “Fetal and neonatal outcome measures were: fetal growth parameters (head circumference, abdominal circumference, femur length, biparietal diameter, estimated fetal weight calculated by any formula), birth-weight (g or kg), LGA (birth-weight >90^th^ centile for gestational age, macrosomia (birth-weight >4kg), neonatal ponderal index (kg/m^3^), neonatal abdominal, head, chest, and waist circumferences (cm), neonatal skinfold thicknesses (mm) and…” | Methods and Materials (Study selection and data extraction). Paragraph 18. |
| Risk of bias in individual studies | 12 | Describe methods used for assessing risk of bias of individual studies (including specification of whether this was done at the study or outcome level), and how this information is to be used in any data synthesis. “Each study was independently assessed by two authors (JLA and CEA) for quality and validity using the Cochrane Collaboration tool for assessing risk of bias. Seven risk of bias domains were assessed for each study and each domain was given a rating of low risk, unknown risk or high risk of bias (S2 Table). All risk of bias analysis was conducted at the study level”. | Methods and Materials (Quality assessment of included studies). Paragraph 19. |
| Summary measures | 13 | State the principal summary measures (e.g., risk ratio, difference in means). “The principle summary measures utilised in this systematic review were unadjusted odds ratios (OR) (for dichotomous data) or differences in means (for continuous data). | Methods and Materials (Quality assessment of included studies). Paragraph 20. |
| Synthesis of results | 14 | Describe the methods of handling data and combining results of studies, if done, including measures of consistency (e.g., I^2^) for each meta-analysis. “Meta-analysis was performed using Review Manager (RevMan) Version 5.3, Copenhagen: The Nordic Cochrane Centre, the Cochrane Collaboration, 2014) and the ‘*metafor*’ package in R version 3.5.1 [33]. Funnel plots were…” | Methods and Materials (Quality assessment of included studies). Paragraph 20. |

| Risk of bias across studies | 15 | Specify any assessment of risk of bias that may affect the cumulative evidence (e.g., publication bias, selective reporting within studies). “Funnel plots were constructed to assess publication bias. Meta-analyses with 5 or more studies included were also subjected to Egger’s test.” | Methods and Materials (Quality assessment of included studies). Paragraph 20. |
| --- | --- | --- | --- |
| Additional analyses | 16 | Describe methods of additional analyses (e.g., sensitivity or subgroup analyses, meta-regression), if done, indicating which were pre-specified. “Heterogeneity between studies was assessed using the I-squared statistic, and any outcomes showing significant inter-study heterogeneity were analysed using a random-effects model. Sensitivity analyses were performed using leave-one-out sensitivity testing for individual studies, ‘leave-one-criteria-out’ sensitivity testing for studies grouped according to GDM criteria and ‘leave-one-continent-out’ sensitivity analysis for studies grouped according to geographical location [33].” | Methods and Materials (Quality assessment of included studies). Paragraph 20. |
| **RESULTS** | | |  |
| Study selection | 17 | Give numbers of studies screened, assessed for eligibility, and included in the review, with reasons for exclusions at each stage, ideally with a flow diagram. “For the comparisons of drug treatments for gestational diabetes, electronic searching of the specified data-bases yielded a total of 3373 studies. After removal of duplicates and title/abstract screening, 157 trials were screened for full text assessment, applying the full set of eligibility criteria. After full-text evaluation, a total of 40 studies remained eligible for inclusion. Seven studies were removed due to not being analysed on an intention-to-treat basis, leaving 33 studies for meta-analysis (Fig 1)...” | Results, Paragraph 21. |
| Study characteristics | 18 | For each study, present characteristics for which data were extracted (e.g., study size, PICOS, follow-up period) and provide the citations.  “For all comparisons, the studies varied with respect to quality and design (S3 Table). Measured outcomes varied between studies and comparisons, with birth-weight the single most commonly reported outcome (Table 1). The doses of glyburide (1.25mg to 20mg daily) and metformin (500mg to 3000mg daily) demonstrated considerable heterogeneity, both within and between studies. Heterogeneity also existed between studies in criteria used to diagnose GDM, with a total of 9 different diagnostic criteria used. These were the ADA, ADIPS, Brazilian Health Ministry (BHM), Carpenter-Coustan (CC), Finnish National Criteria (FNC), International Association of Diabetes and Pregnancy (IADPSG), National Diabetes Data Group (NDDG) and World Health Organisation (WHO) and unspecified GDM criteria. LOO sensitivity analysis demonstrated that use of different thresholds for GDM diagnosis did not have a significant impact on the meta-analyses for birth-weight (S1 Fig). There was also a range of geographical settings including Europe [34-39], USA/Latin America [40-43], Australia/New Zealand [44-46], South America [47-51], North Africa/Middle East [30-32, 52-55], and South East Asia [56-59]. More studies from the North Africa/Middle East [30, 31, 52-55] and Europe [34-38] compared metformin with insulin. More studies from USA/Latin America [40, 41, 43, 47-50] compared glyburide with either insulin or metformin…” | Results, Paragraphs 21 & 22. |
| Risk of bias within studies | 19 | Present data on risk of bias of each study and, if available, any outcome level assessment (see item 12).  “The risk of bias was moderate-to-low in the majority of included studies. However, six studies did not analyse data on an intention-to-treat basis (i.e. trial participants who did not achieve adequate glycaemic control with metformin were removed from the study (22-24, 26, 28, 32), leading to a high risk of bias). A further study had significant imbalance in the baseline characteristics of participants, potentially due to failure of randomisation (39). We performed sub-group meta-analyses, excluding the studies assessed as having a high risk of bias...” | Results, Paragraph 23. |
| Results of individual studies | 20 | For all outcomes considered (benefits or harms), present, for each study: (a) simple summary data for each intervention group (b) effect estimates and confidence intervals, ideally with a forest plot. “In seven studies including 1651 participants [32, 39-41, 47, 48, 58], neonates exposed to glyburide were significantly heavier at birth (58.20g; 95% CI: 10.10g to 106.31g; I_2_=43%, p=0.02) compared to those born to mothers treated with insulin (Fig 3)…” | Results, Paragraphs 22-36. Figs 2- 5. Suppl Fig 5, Tables 1 & 2. |
| Synthesis of results | 21 | Present results of each meta-analysis done, including confidence intervals and measures of consistency. “Three studies [40, 41, 47] including 523 women recorded total gestational weight gain in glyburide compared to insulin-treated women. No difference in total gestational weight gain was observed between glyburide vs. insulin-treated women (-0.68 kg; 95% CI: -1.69 kg to 0.34 kg; I_2_=0%, p=0.19) (Fig 2). Five studies…” | Results, Paragraphs 22-39. |
| Risk of bias across studies | 22 | Present results of any assessment of risk of bias across studies (see Item 15). “Funnel plots for all outcomes were assessed visually for asymmetry (S4 Fig). Egger’s testing demonstrated no evidence of publication bias in any outcomes or comparisons, with the exception of birth-weight for studies comparing glyburide with insulin and for FBS when comparing metformin with insulin”. | Results, Paragraph 24. |
| Additional analysis | 23 | Give results of additional analyses, if done (e.g., sensitivity or subgroup analyses, meta-regression [see Item 16]). “Sensitivity analyses were performed using leave-one-out sensitivity testing for individual studies, ‘leave-one-criteria-out’ sensitivity testing for studies grouped according to GDM criteria and ‘leave-one-continent-out’ sensitivity analysis for studies grouped according to geographical location [33]”. | Results, Paragraph 23. |
| **DISCUSSION** | | |  |
| Summary of evidence | 24 | Summarize the main findings including the strength of evidence for each main outcome; consider their relevance to key groups (e.g., healthcare providers, users, and policy makers). “Our findings show significant differences in neonatal body weight and anthropometric parameters between babies whose mothers were randomised to glyburide, metformin, and insulin to treat GDM, even with equivalent maternal glycaemic control. Our results highlight the importance of considering the effects of treatment on both mother and baby when managing GDM. The lack of data for inclusion in these meta-analyses is concerning, given that numerous bodies worldwide already endorse the use of oral anti-hyperglycaemic agents as first or second line treatments for GDM [14, 15, 17, 18]. Examining in detail the mechanisms by which the various pharmacological options for GDM treatment may impact the developing fetus in both the short and long term is particularly important, given the increasing incidence of GDM worldwide [1, 2]. Most significantly, the greatest increases in GDM globally are occurring populations in which treating GDM with insulin is unlikely to be feasible for large numbers of women [71]. Further understanding of how oral anti-hyperglycaemic agents impact on fetal growth trajectory and later life outcomes should therefore be a research priority”. | Discussion (Strengths, paragraph 43. (Interpretation and Implications) paragraphs 46-47). |
| Limitations | 25 | Discuss limitations at study and outcome level (e.g., risk of bias), and at review-level (e.g., incomplete retrieval of identified research, reporting bias). “The ability to draw definitive conclusions from our meta-analysis is limited by both the quantity and quality of the studies available. With respect to glyburide in particular, few studies met the inclusion criteria (8 studies comparing glyburide to insulin, *vs*. 22 studies comparing metformin to insulin). Moreover, our comparisons of metformin *vs.* glyburide treatment are based on relatively sparse data relating to only 421 women (3 studies). Our findings highlight the surprising lack of high-quality data on which to base clinical recommendations regarding oral anti-hyperglycaemic agents in the treatment of GDM. None of the eligible studies reported the effects of pharmacological intervention for GDM on fetal growth outcomes. Clearly, weight and body composition at birth are products of fetal growth *in utero*, and without fetal data it is challenging to deduce what underlying mechanisms may be driving the observed neonatal outcomes. In light of our findings, there is an urgent need to report fetal growth data in future studies. Similarly..” | Discussion (Limitations), paragraphs 44-45. |
| Conclusions | 26 | Provide a general interpretation of the results in the context of other evidence, and implications for future research. “Our findings show significant differences in neonatal body weight and anthropometric parameters between babies whose mothers were randomised to glyburide, metformin, and insulin to treat GDM, even with equivalent maternal glycaemic control. Our results highlight the importance of considering the effects of treatment on both mother and baby when managing GDM”. | Discussion (Interpretation and Implications) paragraphs 46-47). |
| **FUNDING** | | |  |
| Funding | 27 | Describe sources of funding for the systematic review and other support (e.g., supply of data); role of funders for the systematic review. Funding sources not required in manuscript. This is provided in the financial disclosure section of the manuscript submission system, as requested by the Journal. | N/A |

*From:*  Moher D, Liberati A, Tetzlaff J, Altman DG, The PRISMA Group (2009). Preferred Reporting Items for Systematic Reviews and Meta-Analyses: The PRISMA Statement. PLoS Med 6(7): e1000097. doi:10.1371/journal.pmed1000097

For more information, visit: **www.prisma-statement.org**.

Page 4 of 4

Table 1: PRISMA 2009 Checklist.
