# Supplementary figures and images for: Comparative impact of pharmacological treatments for gestational diabetes on neonatal anthropometry independent of maternal glycaemic control: A systematic review and meta-analysis
Source: PLoS Med. 2020 May 22;17(5):e1003126. doi: 10.1371/journal.pmed.1003126 (PMC7244100; doi:10.1371/journal.pmed.1003126)

## Slide 1
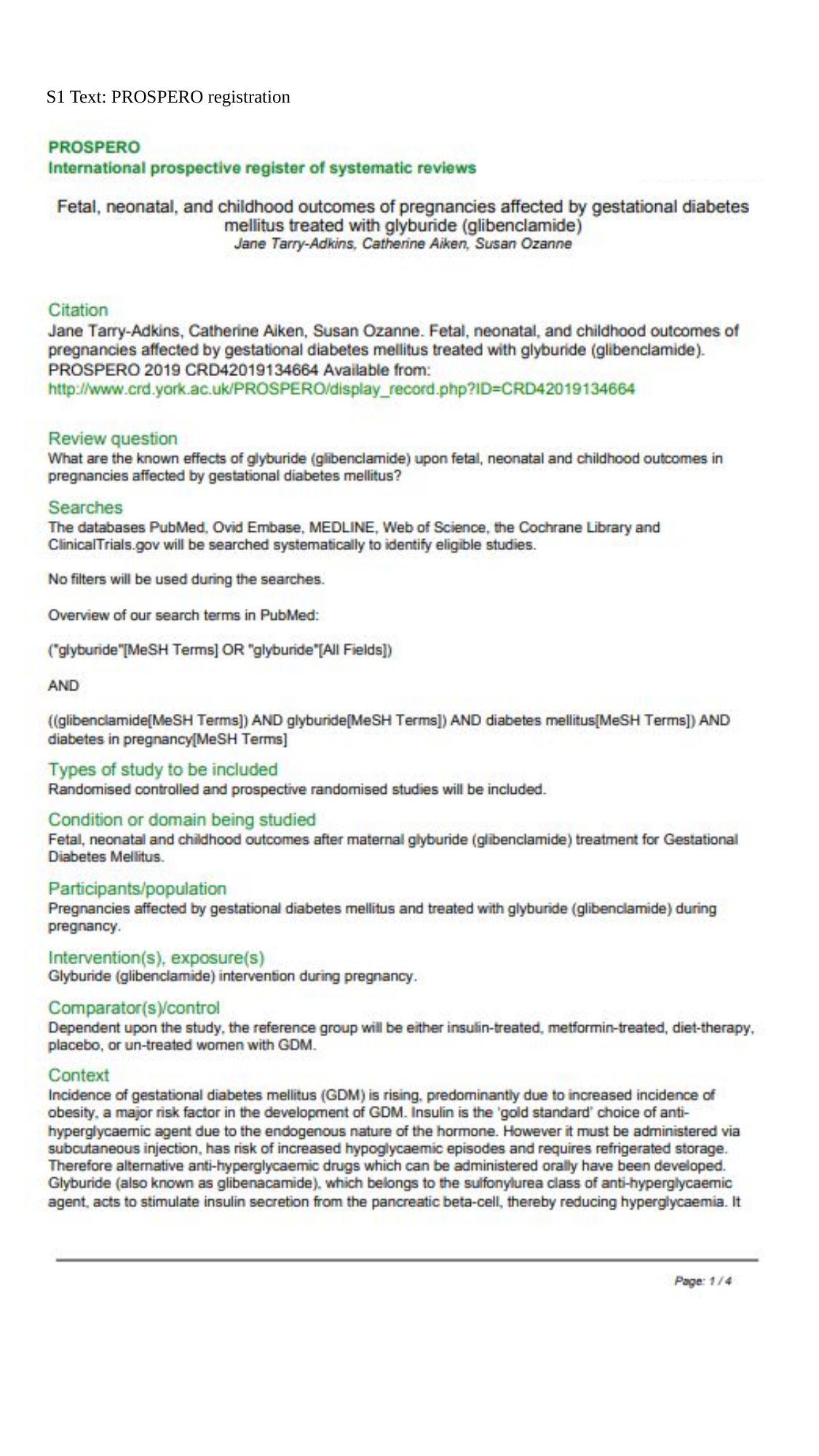

S1 Text: PROSPERO registration

## Slide 2
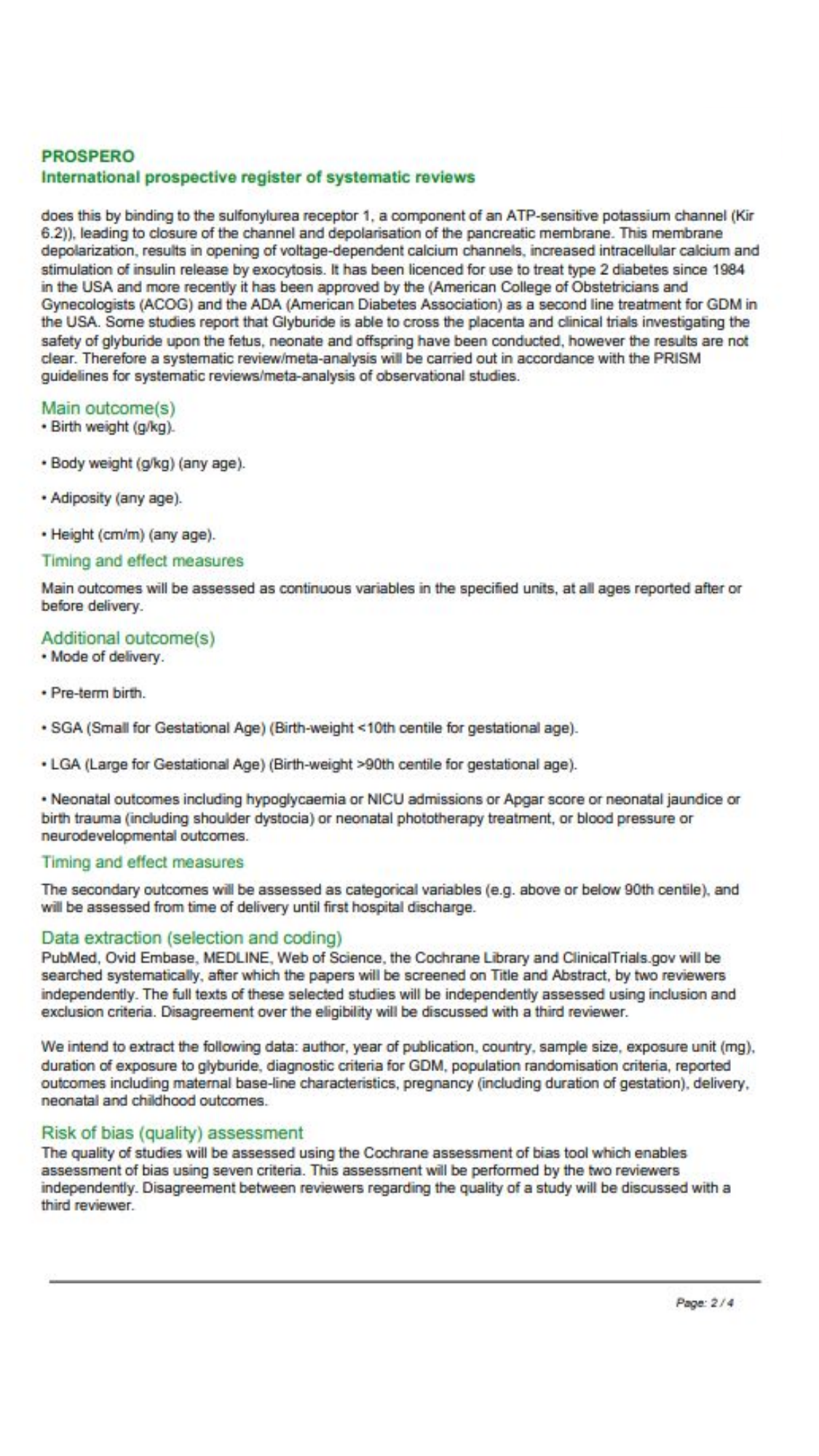

## Slide 3
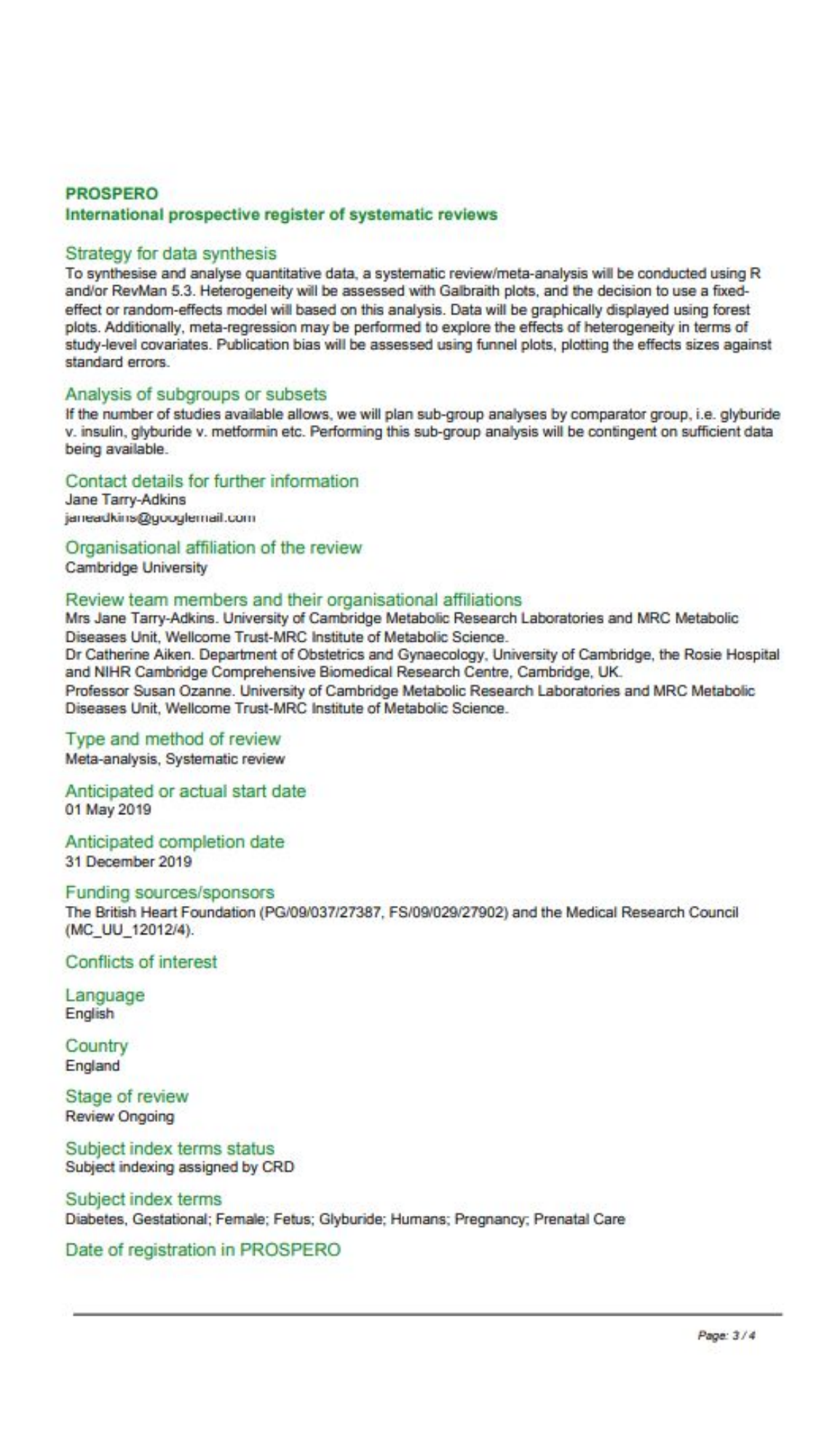

## Slide 4
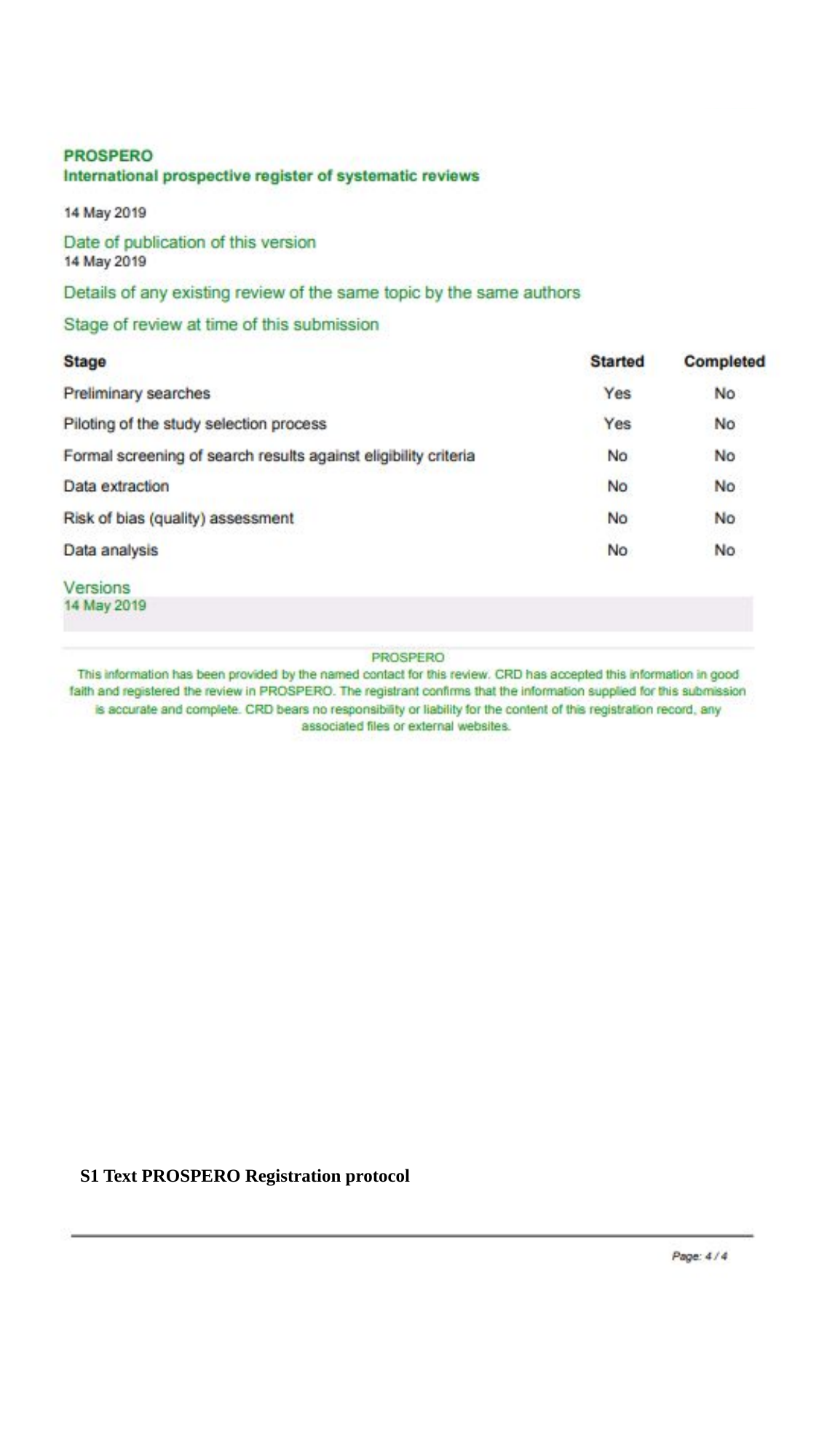

S1 Text PROSPERO Registration protocol

Supplement: S1 Text — CRD42019134664. (PPTX) [file pmed.1003126.s002.pptx]

## Slide 1
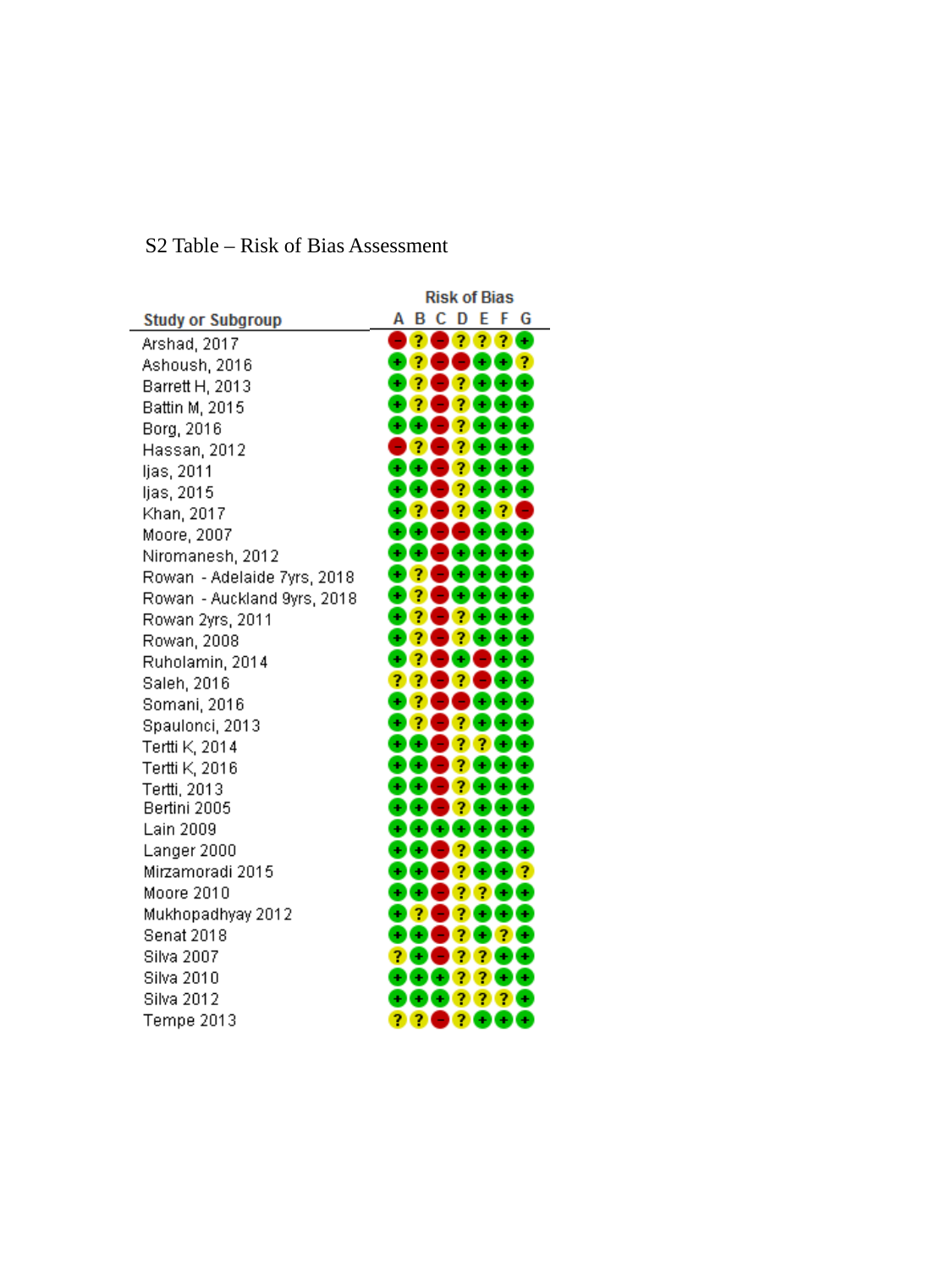

S2 Table – Risk of Bias Assessment

Supplement: S2 Table — (A) Random sequence generation (selection bias), (B) allocation concealment (selection bias), (C) blinding participants and personnel (performance bias), (D) blinding of outcome assessment (detection bias), (E) incomplete outcome data (attrition bias), (F) selection bias (reporting bias), and (G) other bias. (PPTX) [file pmed.1003126.s005.pptx]
