# Supplementary material for: Comparative impact of pharmacological treatments for gestational diabetes on neonatal anthropometry independent of maternal glycaemic control: A systematic review and meta-analysis
Source: PLoS Med. 2020 May 22;17(5):e1003126. doi: 10.1371/journal.pmed.1003126 (PMC7244100; doi:10.1371/journal.pmed.1003126)
Supplement: S2 Text — (A) PubMed, (B) OVID EMBASE, (C) Medline, (D) Web of Science, (E) Cochrane Library, and (F) www.clinicaltrials.gov. (PPTX) [file pmed.1003126.s003.pptx]

## Slide 1
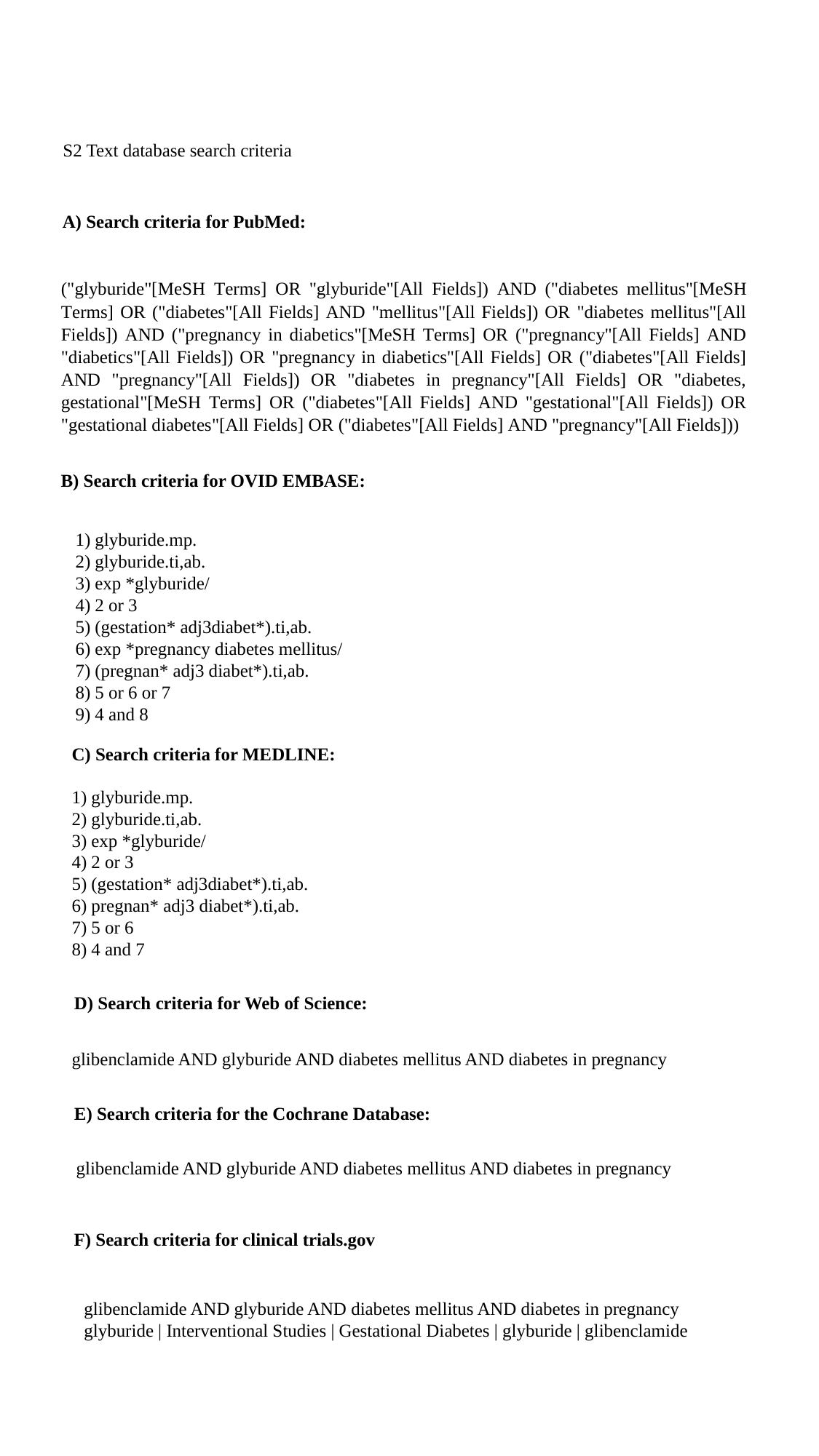

S2 Text database search criteria
A) Search criteria for PubMed:
B) Search criteria for OVID EMBASE:
1) glyburide.mp.
2) glyburide.ti,ab.
3) exp *glyburide/
4) 2 or 3
5) (gestation* adj3diabet*).ti,ab.
6) exp *pregnancy diabetes mellitus/
7) (pregnan* adj3 diabet*).ti,ab.
8) 5 or 6 or 7
9) 4 and 8
C) Search criteria for MEDLINE:
1) glyburide.mp.
2) glyburide.ti,ab.
3) exp *glyburide/
4) 2 or 3
5) (gestation* adj3diabet*).ti,ab.
6) pregnan* adj3 diabet*).ti,ab.
7) 5 or 6
8) 4 and 7
D) Search criteria for Web of Science:
glibenclamide AND glyburide AND diabetes mellitus AND diabetes in pregnancy
E) Search criteria for the Cochrane Database:
glibenclamide AND glyburide AND diabetes mellitus AND diabetes in pregnancy
F) Search criteria for clinical trials.gov
glibenclamide AND glyburide AND diabetes mellitus AND diabetes in pregnancy
glyburide | Interventional Studies | Gestational Diabetes | glyburide | glibenclamide
