# Supplementary material for: Comparative impact of pharmacological treatments for gestational diabetes on neonatal anthropometry independent of maternal glycaemic control: A systematic review and meta-analysis
Source: PLoS Med. 2020 May 22;17(5):e1003126. doi: 10.1371/journal.pmed.1003126 (PMC7244100; doi:10.1371/journal.pmed.1003126)
Supplement: S4 Table — (PPTX) [file pmed.1003126.s007.pptx]

## Slide 1
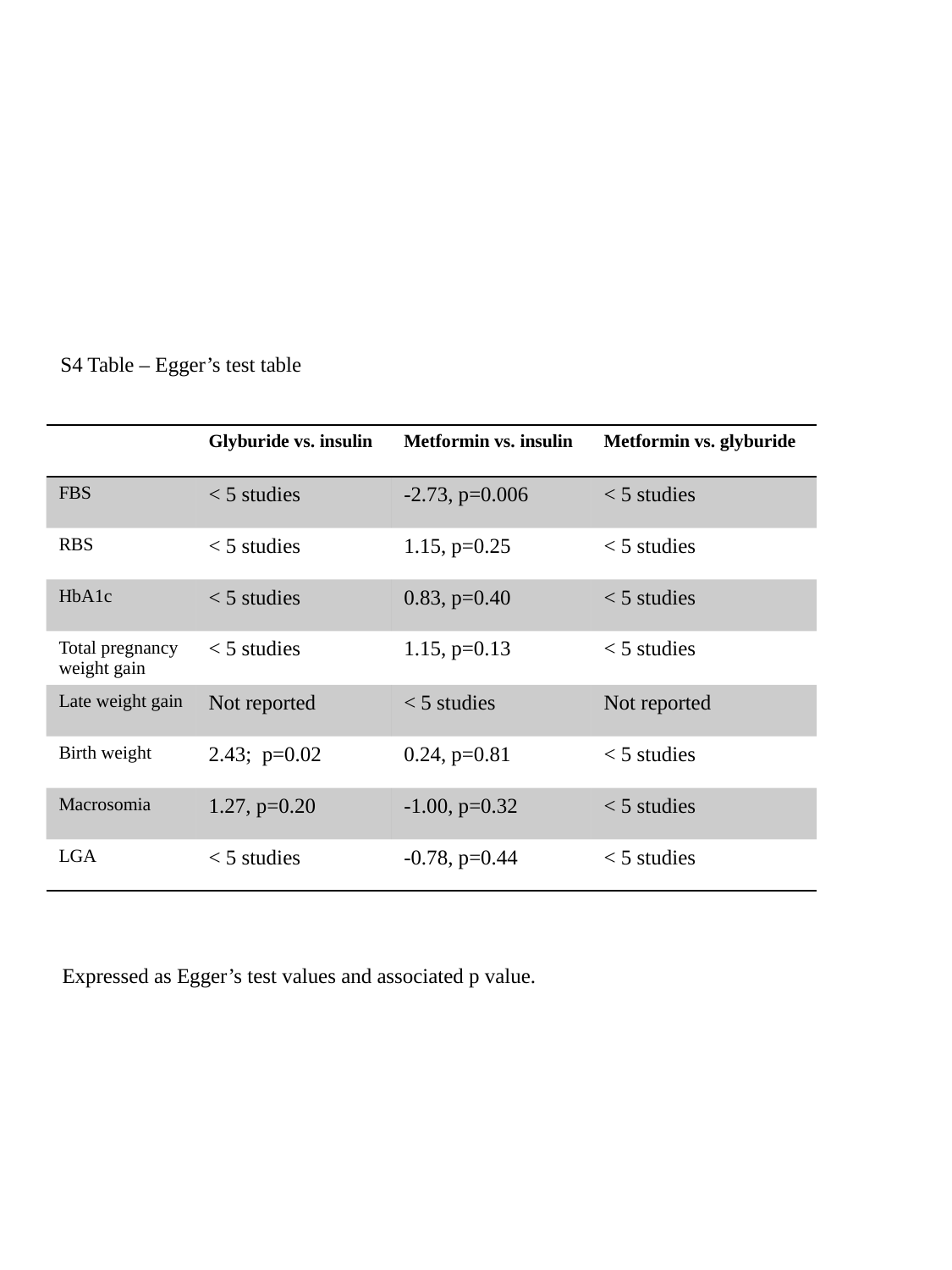

S4 Table – Egger’s test table
| | Glyburide vs. insulin | Metformin vs. insulin | Metformin vs. glyburide |
| --- | --- | --- | --- |
| FBS | < 5 studies | -2.73, p=0.006 | < 5 studies |
| RBS | < 5 studies | 1.15, p=0.25 | < 5 studies |
| HbA1c | < 5 studies | 0.83, p=0.40 | < 5 studies |
| Total pregnancy weight gain | < 5 studies | 1.15, p=0.13 | < 5 studies |
| Late weight gain | Not reported | < 5 studies | Not reported |
| Birth weight | 2.43; p=0.02 | 0.24, p=0.81 | < 5 studies |
| Macrosomia | 1.27, p=0.20 | -1.00, p=0.32 | < 5 studies |
| LGA | < 5 studies | -0.78, p=0.44 | < 5 studies |
Expressed as Egger’s test values and associated p value.
