# Supplementary material for: Comparative impact of pharmacological treatments for gestational diabetes on neonatal anthropometry independent of maternal glycaemic control: A systematic review and meta-analysis
Source: PLoS Med. 2020 May 22;17(5):e1003126. doi: 10.1371/journal.pmed.1003126 (PMC7244100; doi:10.1371/journal.pmed.1003126)
Supplement: S1 Fig — ADA, ADIPS, BHM, CC, FNC, IADPSG, NDDG, WHO, and no criteria detailed. ADA, American Diabetes Association; ADIPS, Australasian Diabetes in Pregnancy Society; BHM, Brazilian Health Ministry; CC, Carpenter-Coustan; FNC, Finnish National Criteria; IADPSG, International Association of Diabetes and Pregnancy Study Groups; NDDG, National Diabetes Data group; WHO, World Health Organization. (PPTX) [file pmed.1003126.s008.pptx]

## Slide 1
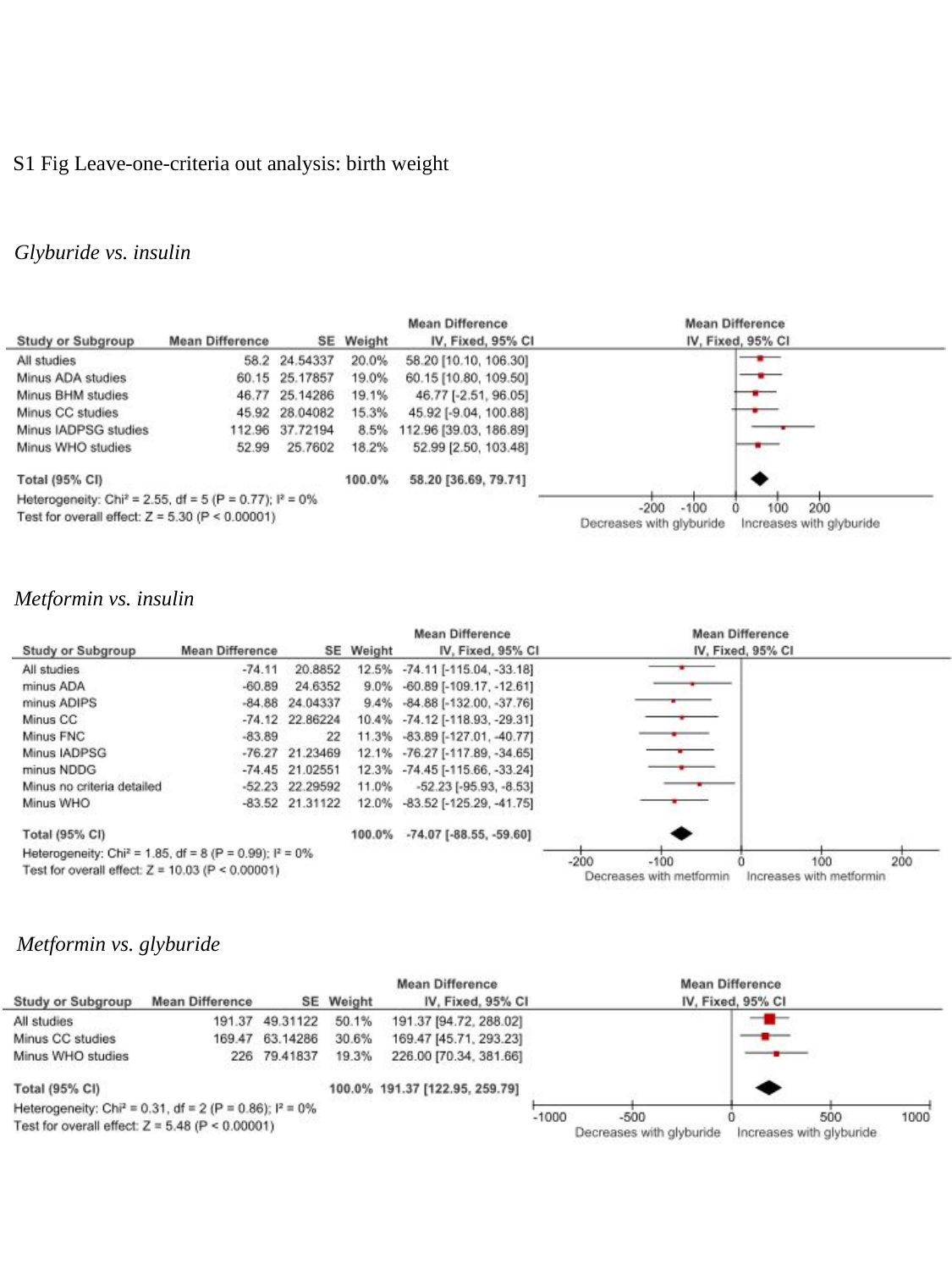

S1 Fig Leave-one-criteria out analysis: birth weight
Glyburide vs. insulin
Metformin vs. insulin
Metformin vs. glyburide
