# Supplementary material for: Comparative impact of pharmacological treatments for gestational diabetes on neonatal anthropometry independent of maternal glycaemic control: A systematic review and meta-analysis
Source: PLoS Med. 2020 May 22;17(5):e1003126. doi: 10.1371/journal.pmed.1003126 (PMC7244100; doi:10.1371/journal.pmed.1003126)
Supplement: S2 Fig — (PPTX) [file pmed.1003126.s009.pptx]

## Slide 1
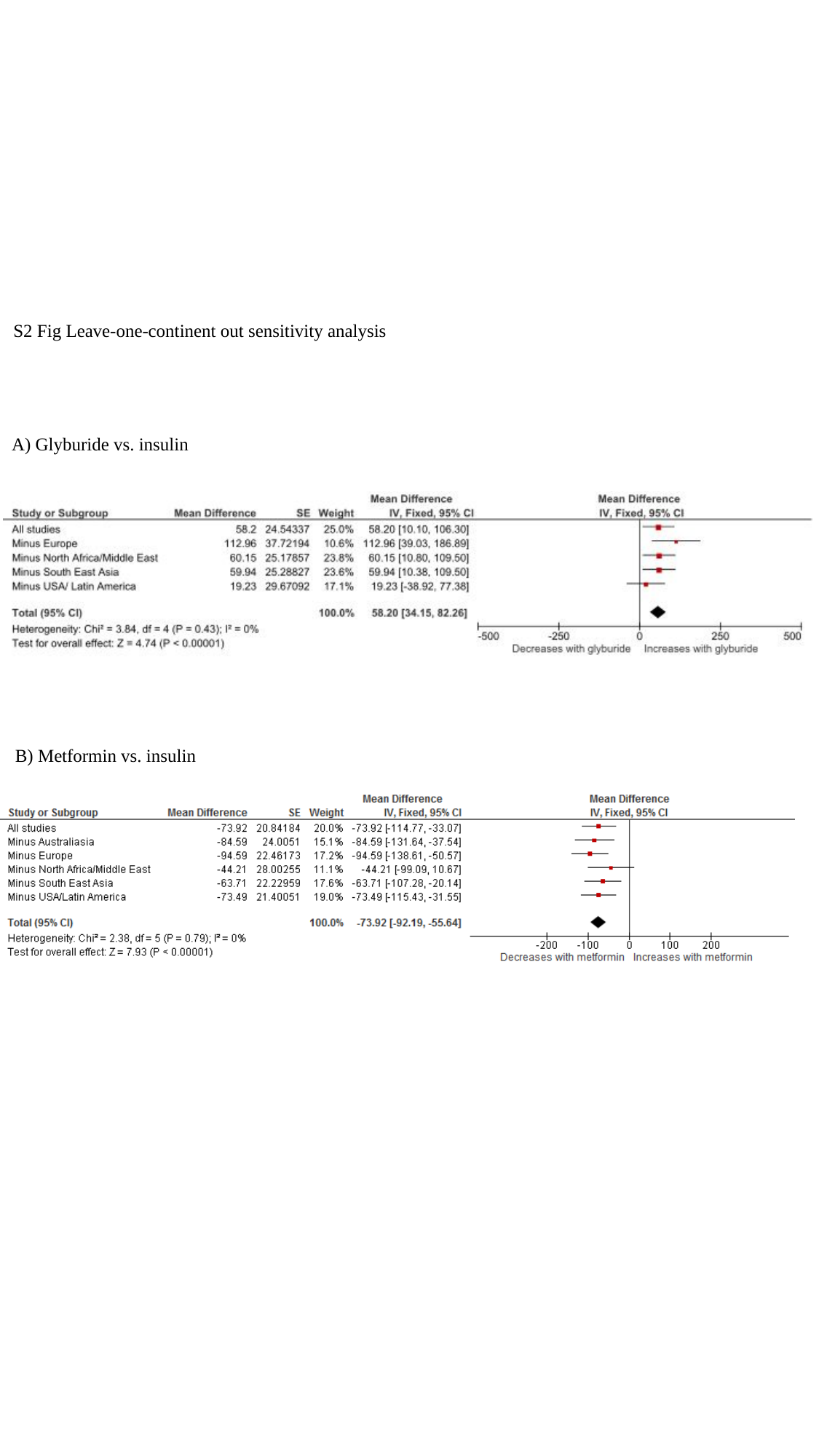

S2 Fig Leave-one-continent out sensitivity analysis
A) Glyburide vs. insulin
B) Metformin vs. insulin
