# Supplementary material for: Comparative impact of pharmacological treatments for gestational diabetes on neonatal anthropometry independent of maternal glycaemic control: A systematic review and meta-analysis
Source: PLoS Med. 2020 May 22;17(5):e1003126. doi: 10.1371/journal.pmed.1003126 (PMC7244100; doi:10.1371/journal.pmed.1003126)
Supplement: S3 Fig — (A) Gestational weight gain, (B) birth weight, (C) macrosomia, (D) LGA, (E) FBS, (F) RBS, and (G) HbA1c in glyburide versus insulin, metformin versus insulin, and metformin versus glyburide comparisons. All outcomes expressed as OR (95% CI). CI, confidence interval; FBS, fasting blood glucose; HbA1c, glycated haemoglobin; LGA, large for gestational age; LOO, leave-one-out; OR, odds ratio; RBS, random blood glucose. (PPTX) [file pmed.1003126.s010.pptx]

## Slide 1
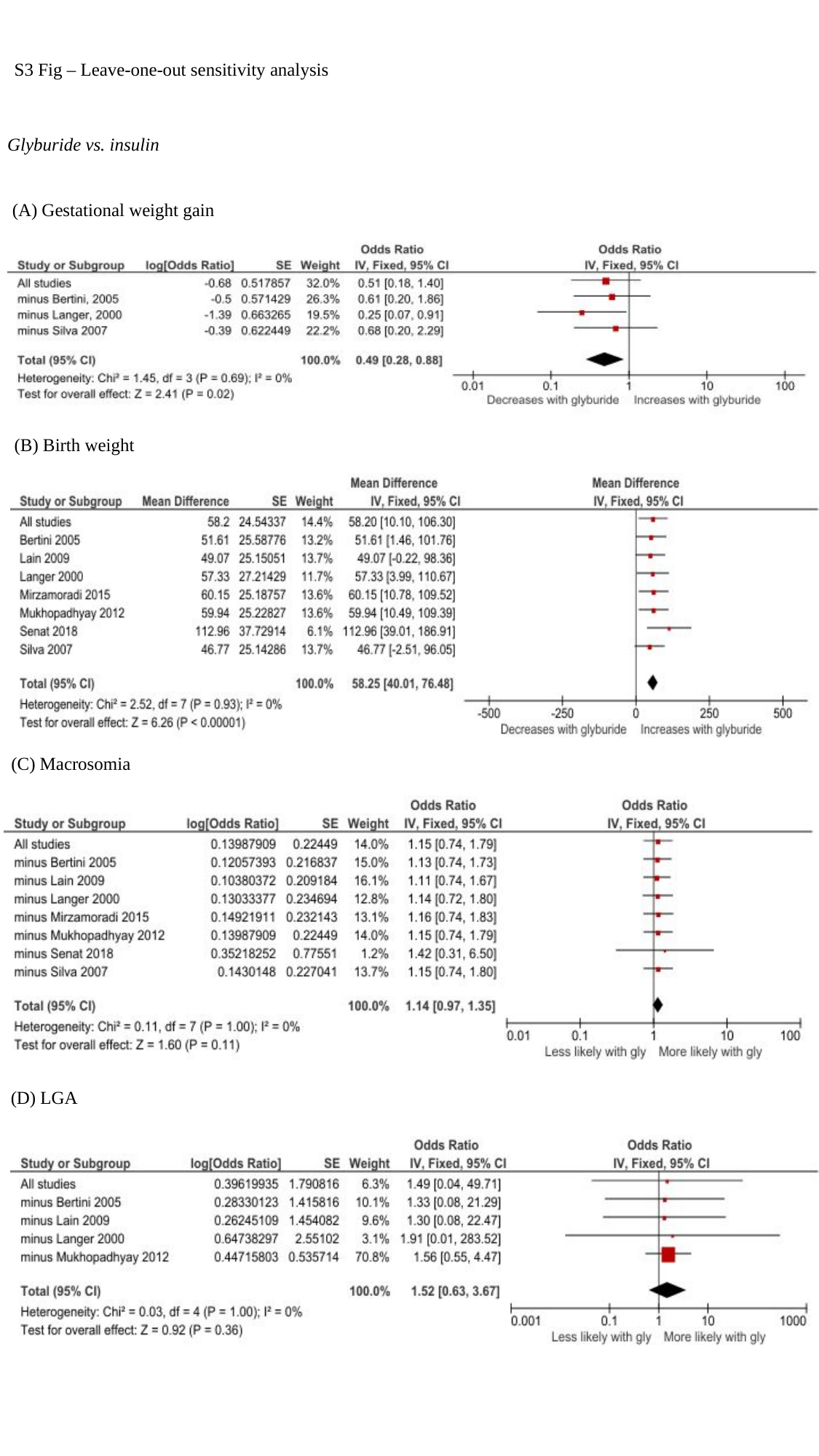

S3 Fig – Leave-one-out sensitivity analysis
Glyburide vs. insulin
(A) Gestational weight gain
(B) Birth weight
(C) Macrosomia
(D) LGA

## Slide 2
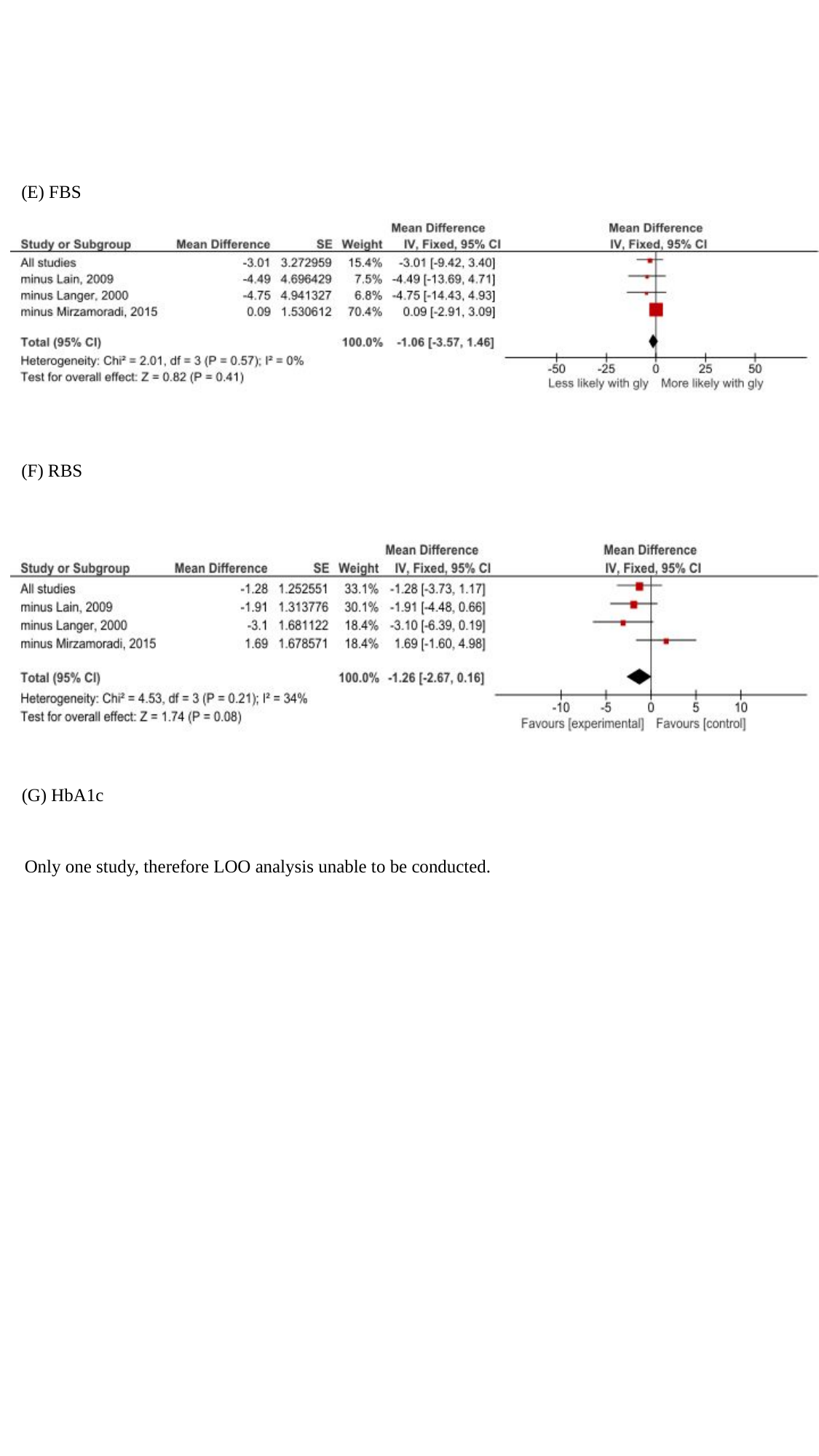

(E) FBS
(F) RBS
(G) HbA1c
Only one study, therefore LOO analysis unable to be conducted.

## Slide 3
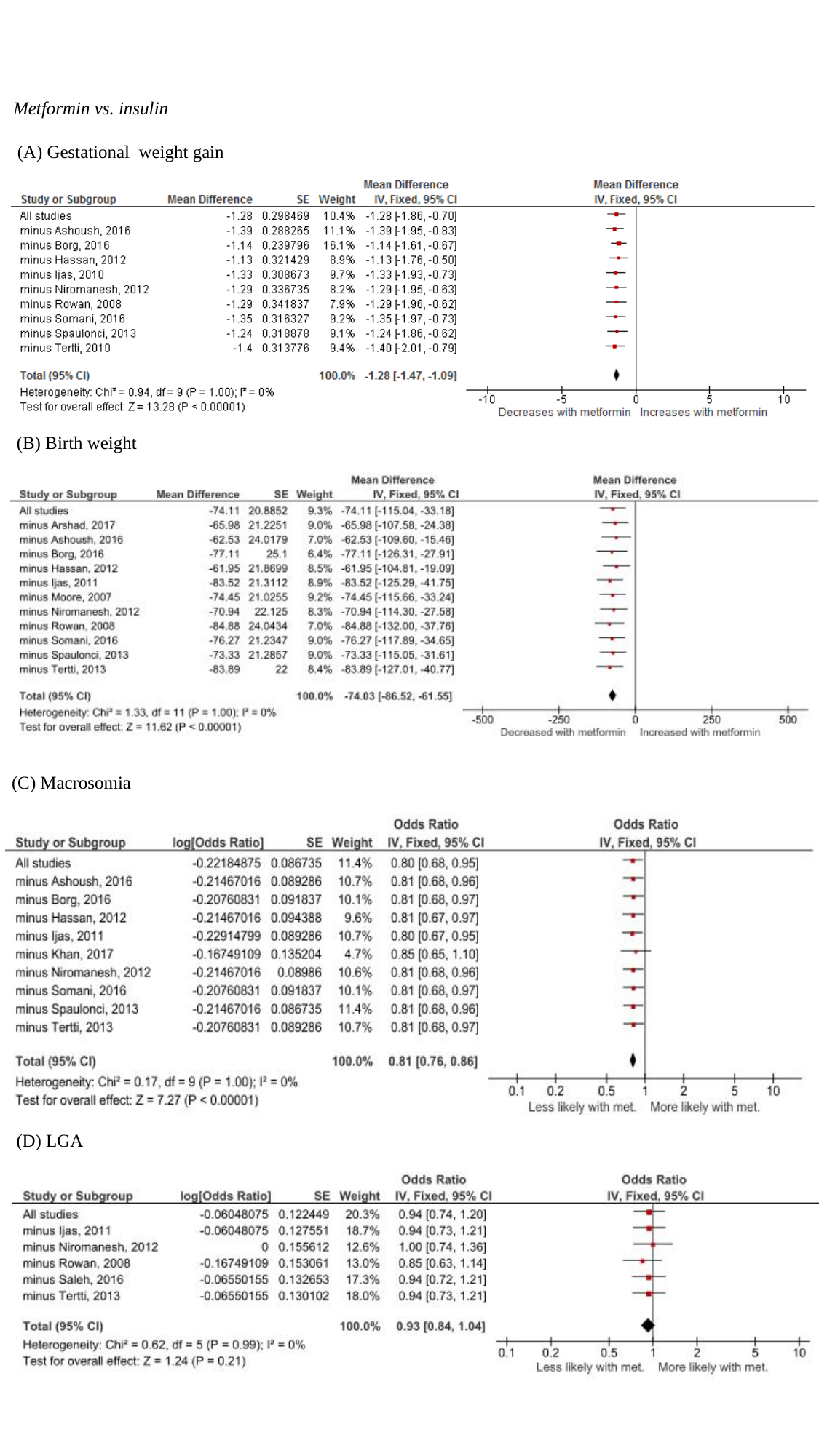

Metformin vs. insulin
(A) Gestational weight gain
(B) Birth weight
(C) Macrosomia
(D) LGA

## Slide 4
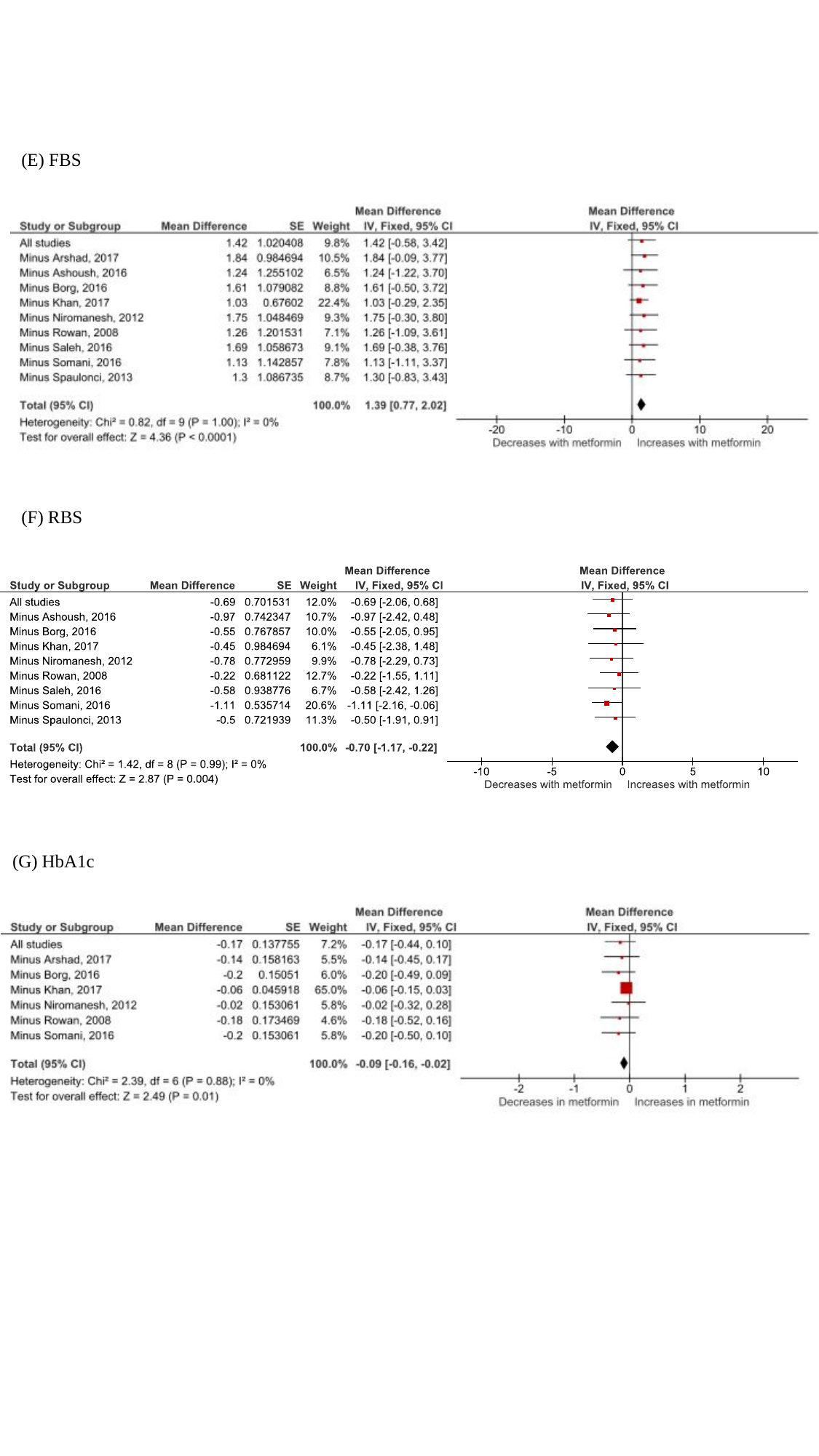

(E) FBS
(F) RBS
(G) HbA1c

## Slide 5
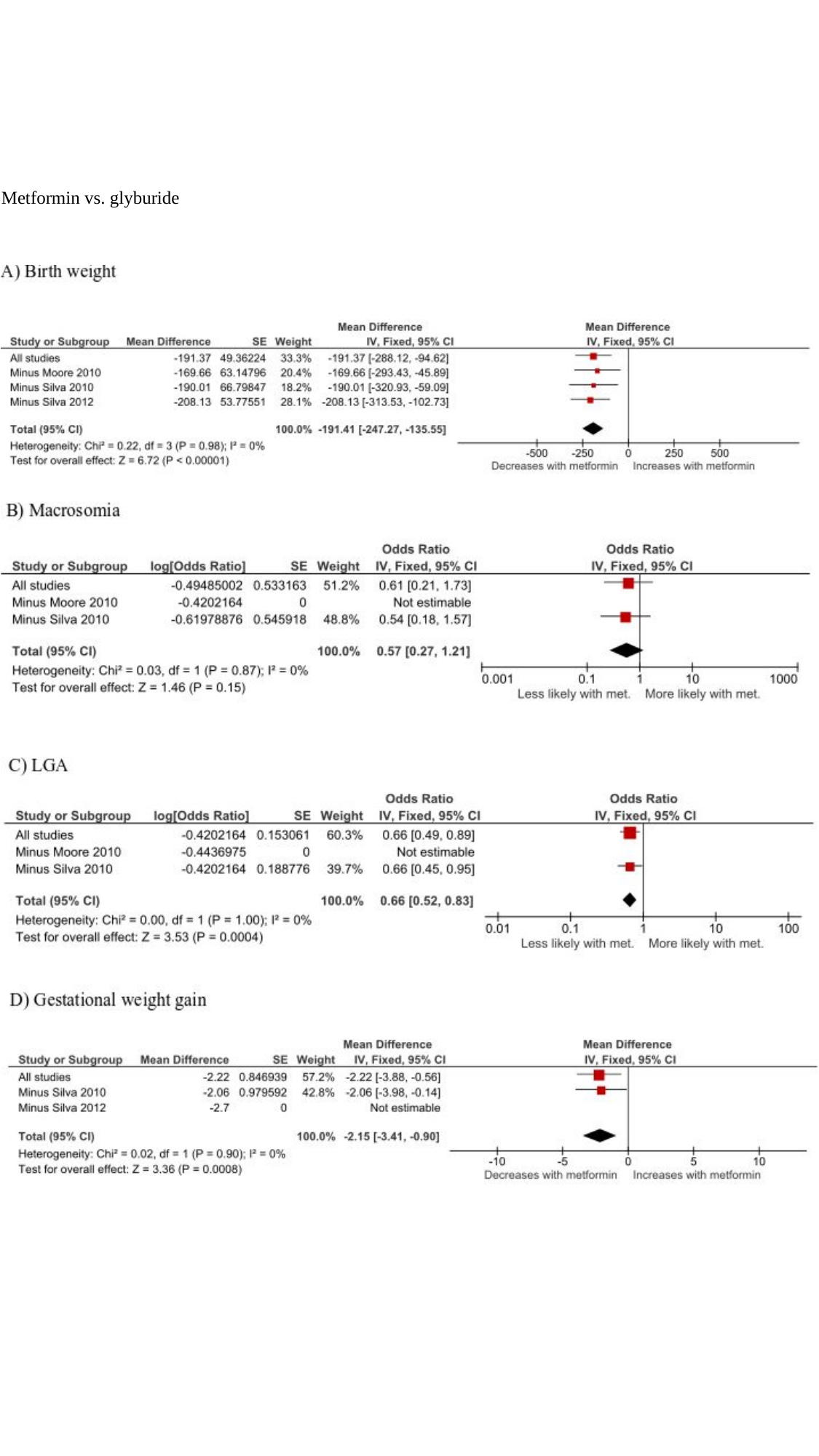

Metformin vs. glyburide

## Slide 6
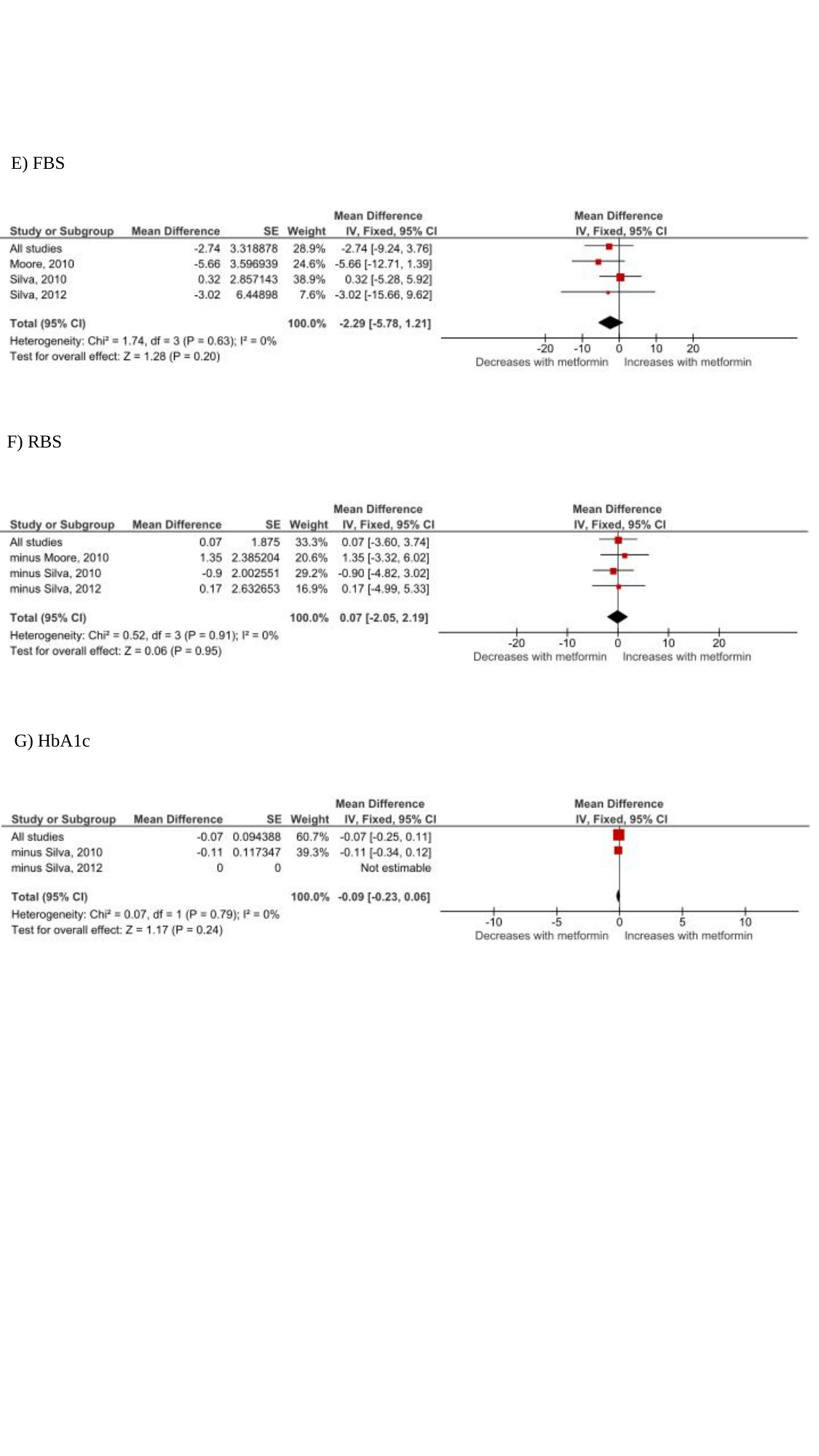

E) FBS
F) RBS
G) HbA1c
