# Supplementary material for: Comparative impact of pharmacological treatments for gestational diabetes on neonatal anthropometry independent of maternal glycaemic control: A systematic review and meta-analysis
Source: PLoS Med. 2020 May 22;17(5):e1003126. doi: 10.1371/journal.pmed.1003126 (PMC7244100; doi:10.1371/journal.pmed.1003126)
Supplement: S4 Fig — All outcomes plotted. (PPTX) [file pmed.1003126.s011.pptx]

## Slide 1
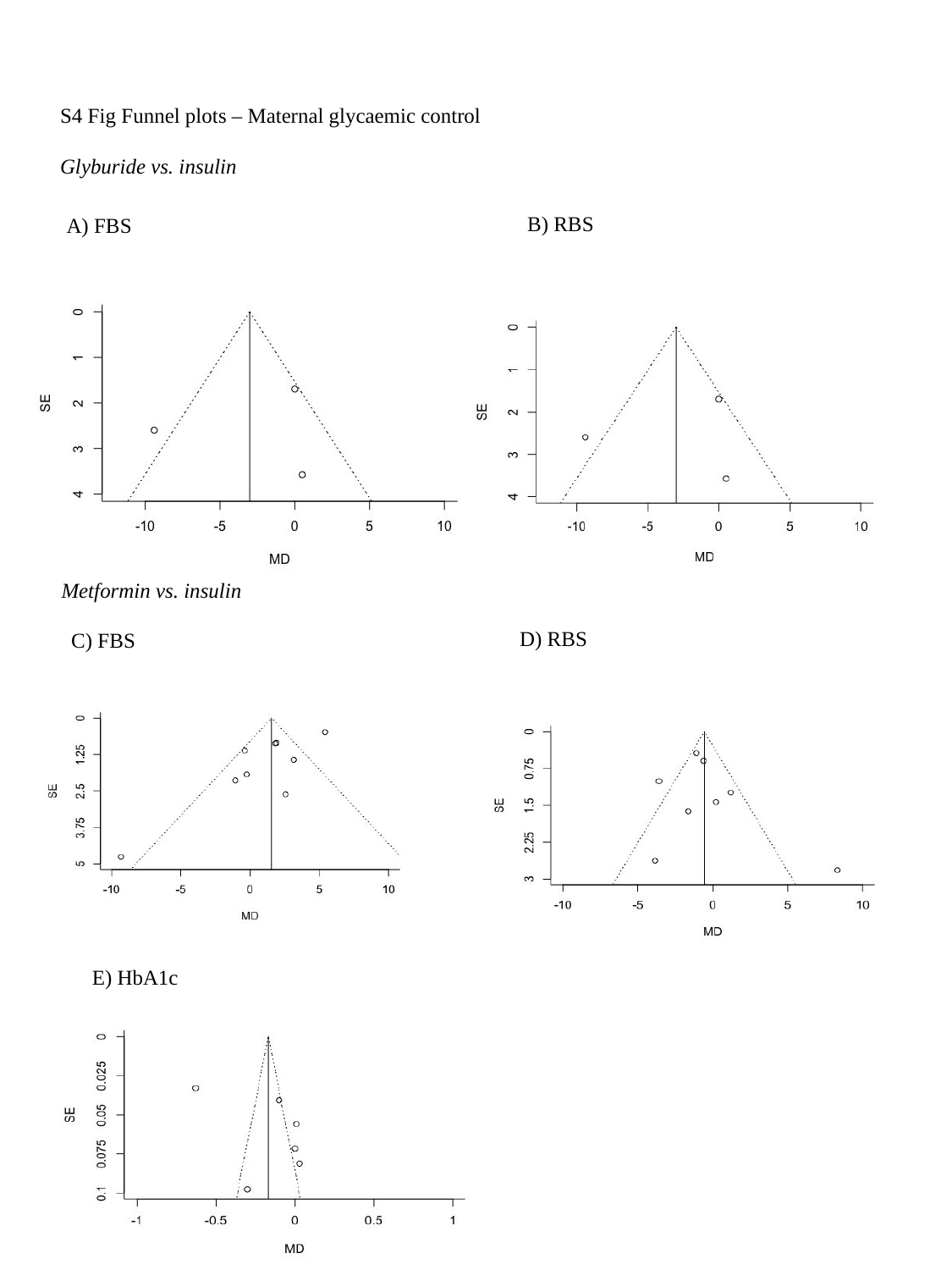

S4 Fig Funnel plots – Maternal glycaemic control
Glyburide vs. insulin
B) RBS
A) FBS
Metformin vs. insulin
D) RBS
C) FBS
E) HbA1c

## Slide 2
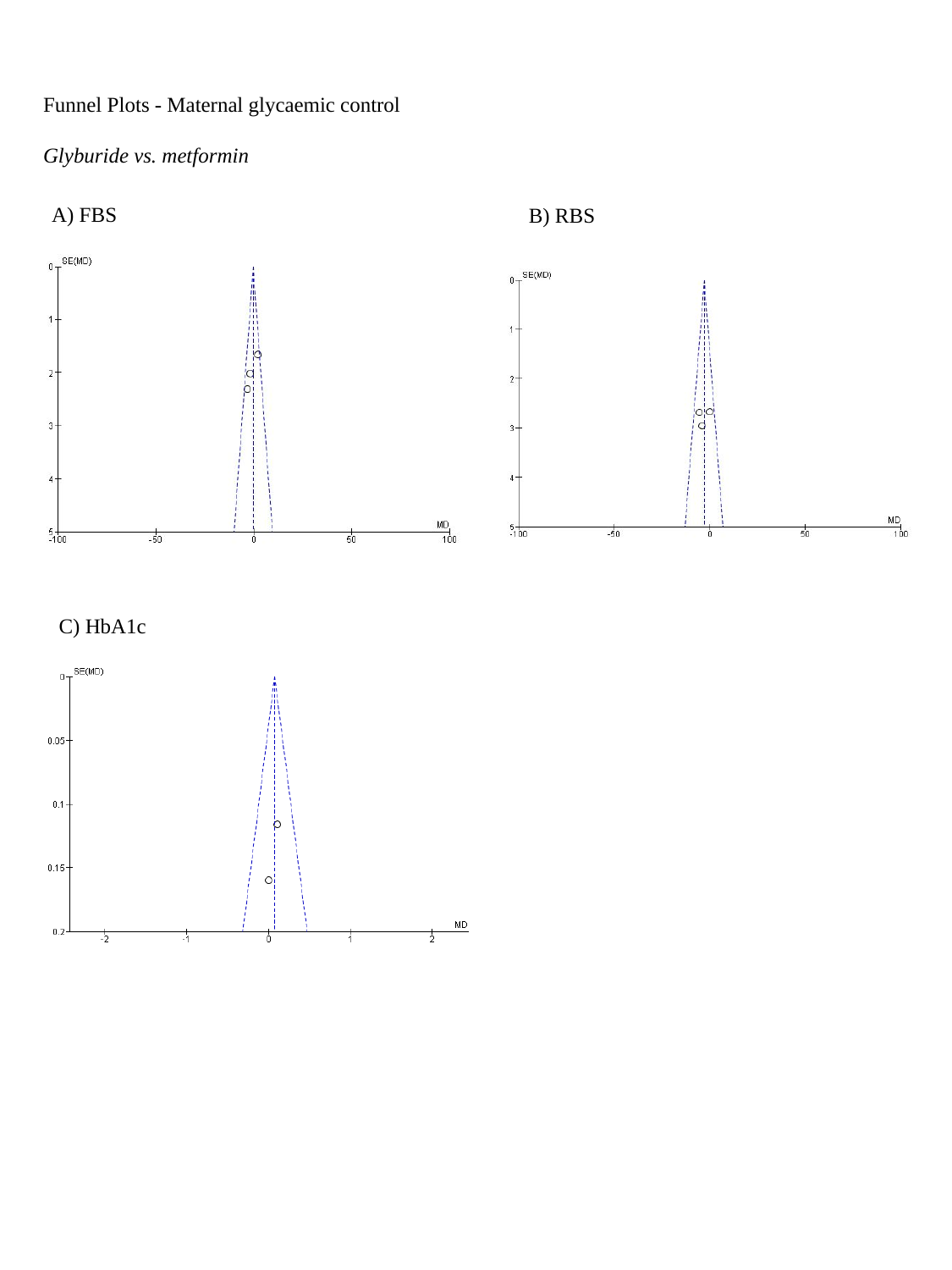

Funnel Plots - Maternal glycaemic control
Glyburide vs. metformin
A) FBS
B) RBS
C) HbA1c

## Slide 3
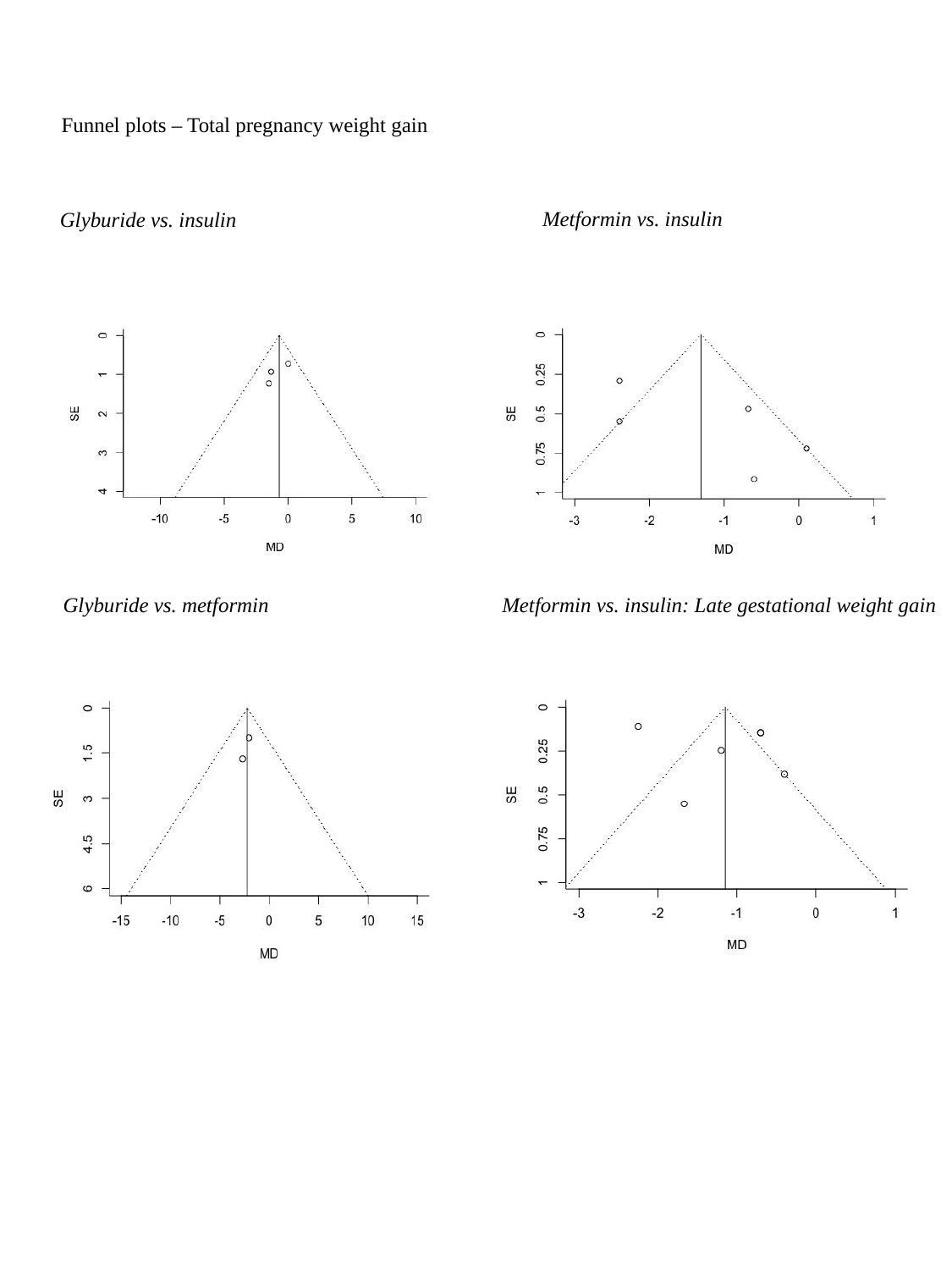

Funnel plots – Total pregnancy weight gain
Metformin vs. insulin
Glyburide vs. insulin
Glyburide vs. metformin
Metformin vs. insulin: Late gestational weight gain

## Slide 4
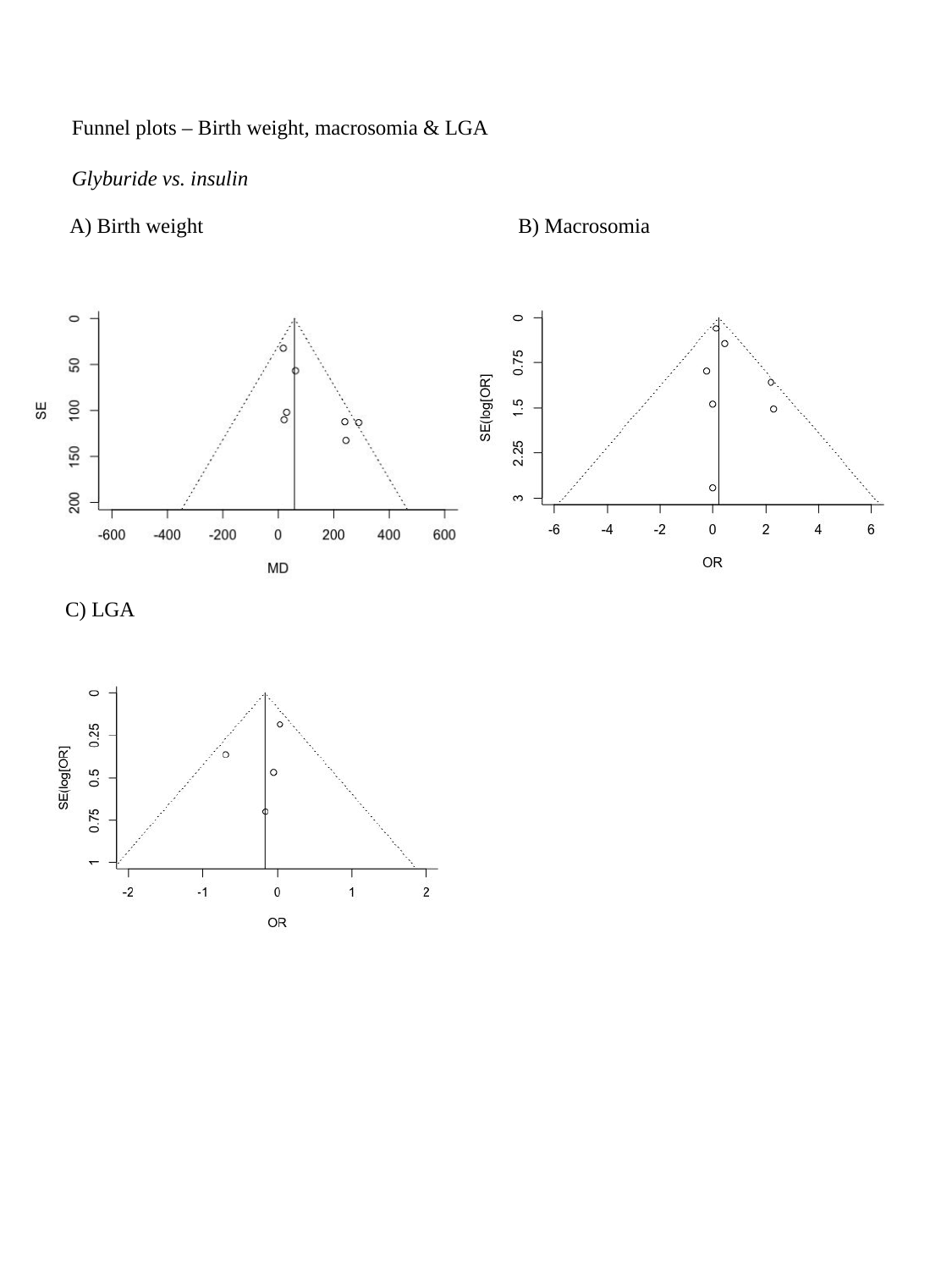

Funnel plots – Birth weight, macrosomia & LGA
Glyburide vs. insulin
B) Macrosomia
A) Birth weight
C) LGA

## Slide 5
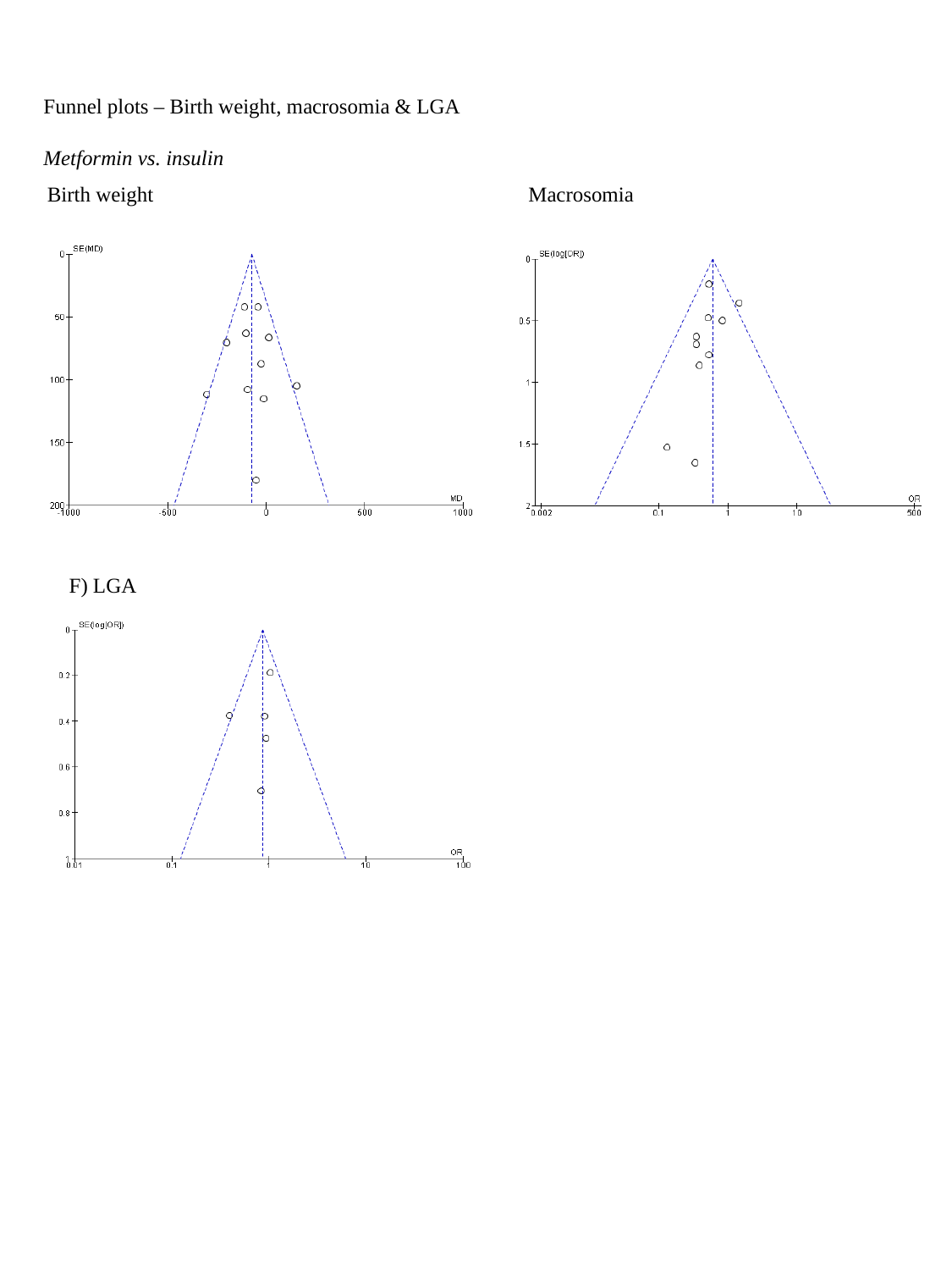

Funnel plots – Birth weight, macrosomia & LGA
Metformin vs. insulin
 Birth weight
Macrosomia
F) LGA

## Slide 6
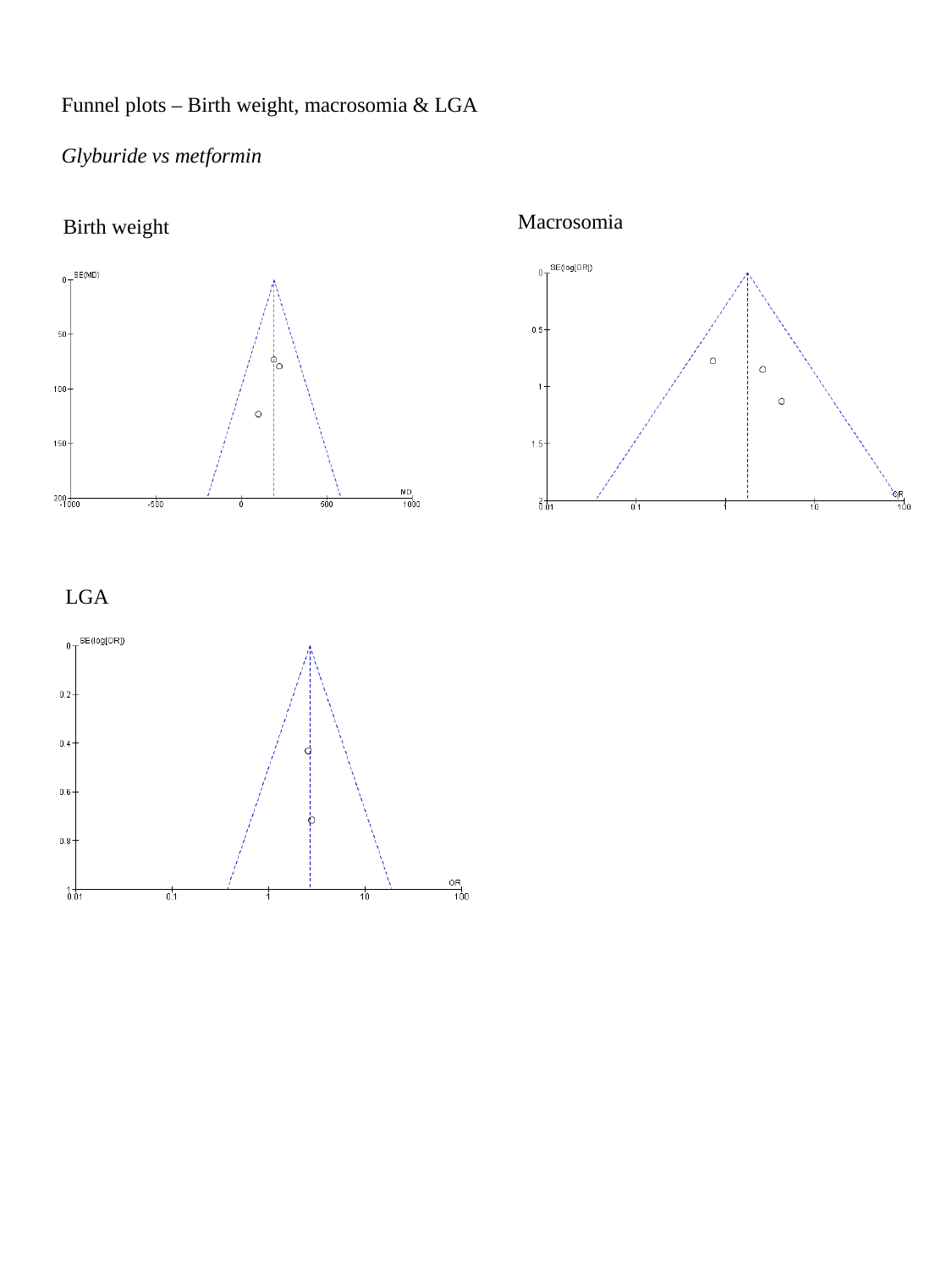

Funnel plots – Birth weight, macrosomia & LGA
Glyburide vs metformin
Macrosomia
Birth weight
LGA
