# Supplementary material for: Comparative impact of pharmacological treatments for gestational diabetes on neonatal anthropometry independent of maternal glycaemic control: A systematic review and meta-analysis
Source: PLoS Med. 2020 May 22;17(5):e1003126. doi: 10.1371/journal.pmed.1003126 (PMC7244100; doi:10.1371/journal.pmed.1003126)
Supplement: S5 Fig — All outcomes and comparisons were N/S. FBS, fasting blood glucose; HbA1c, glycated haemoglobin; RBS, random blood glucose. (PPTX) [file pmed.1003126.s012.pptx]

## Slide 1
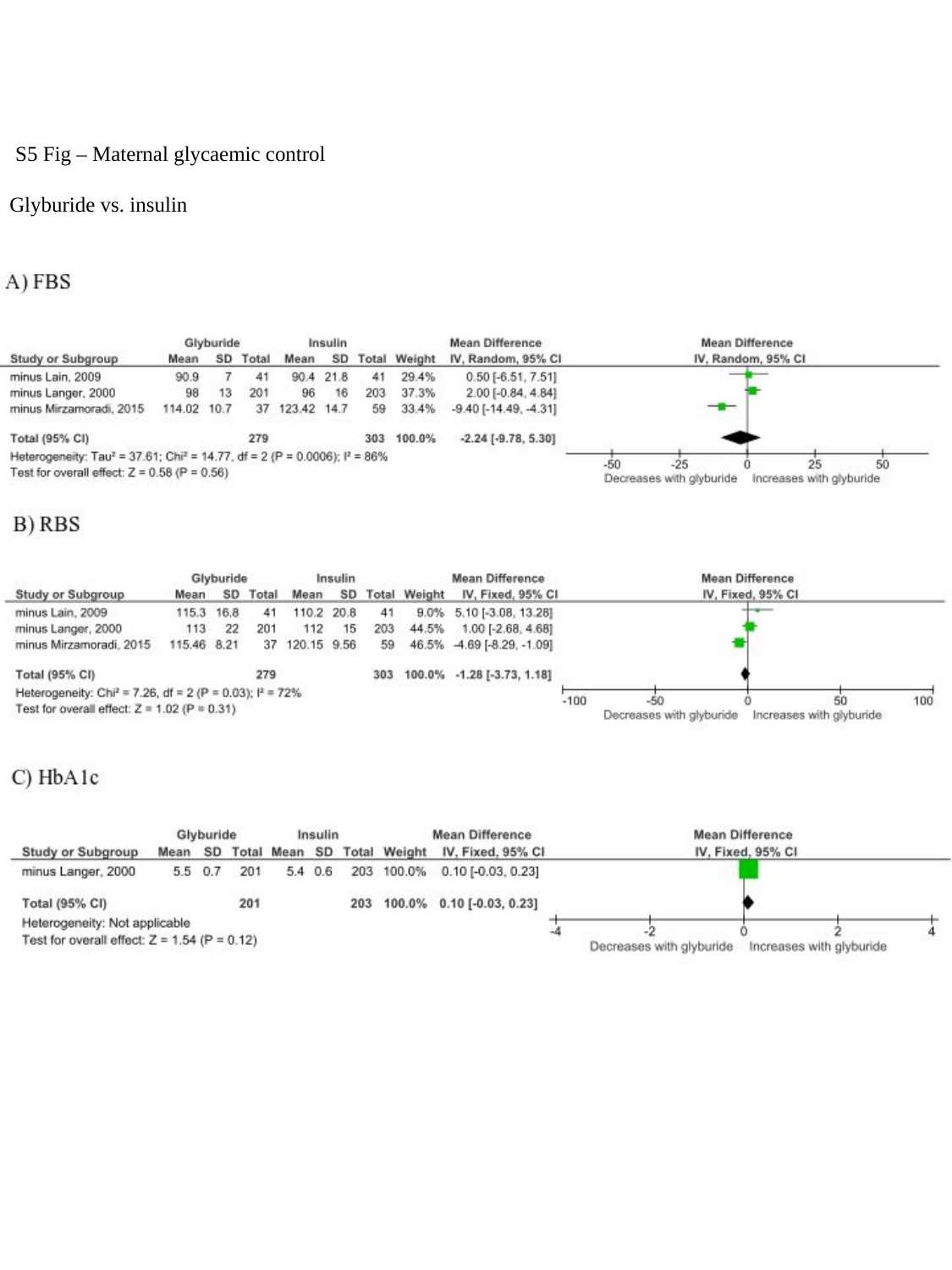

S5 Fig – Maternal glycaemic control
Glyburide vs. insulin

## Slide 2
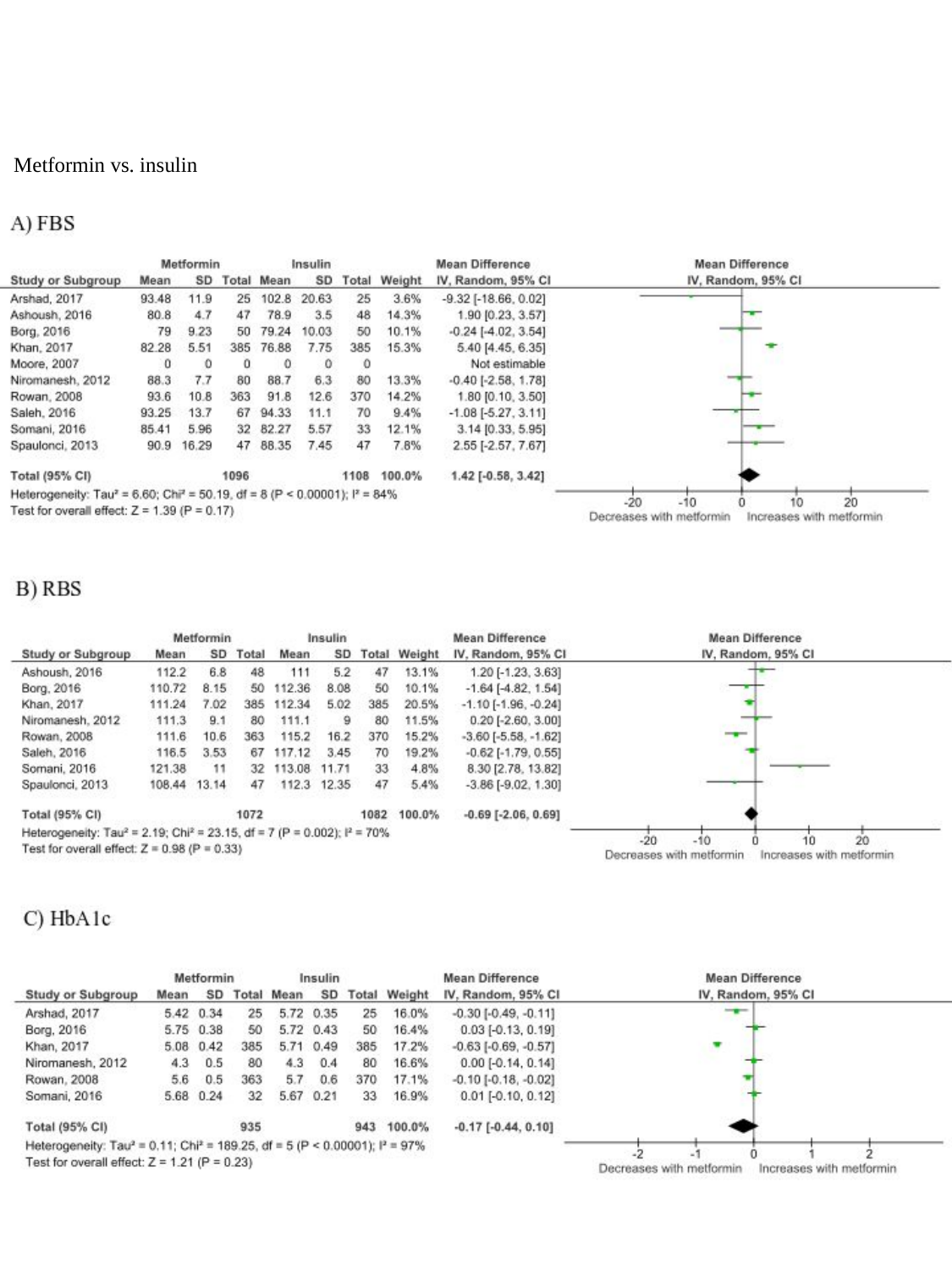

Metformin vs. insulin

## Slide 3
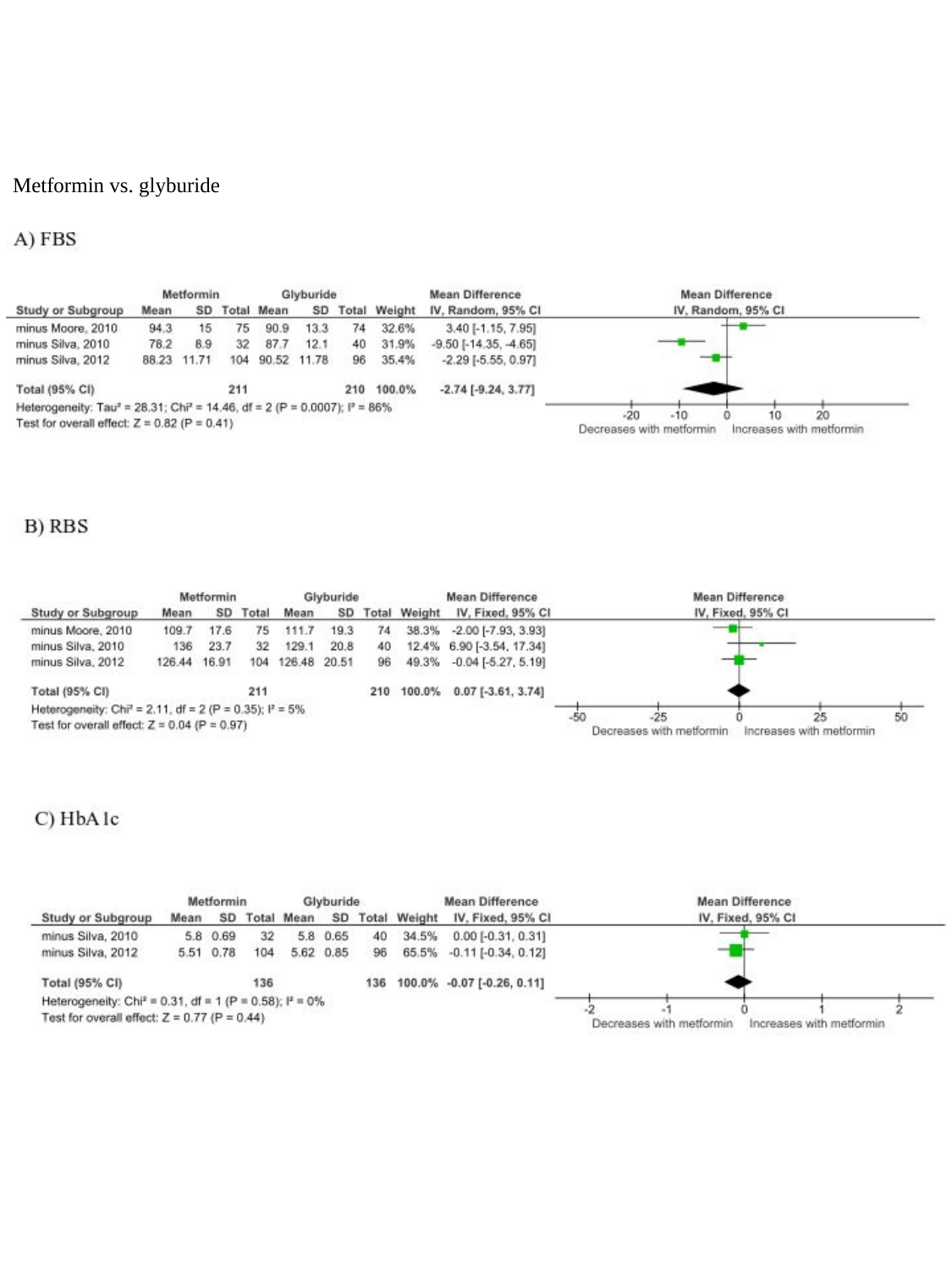

Metformin vs. glyburide
